# Ultrasound elastography of back muscle biomechanical properties: a systematic review and meta-analysis of current methods

**DOI:** 10.1186/s13244-024-01785-7

**Published:** 2024-08-14

**Authors:** Mercedes David, Karine Devantéry, Bénédicte Nauche, Miguel Chagnon, Mark Keezer, Nathaly Gaudreault, Nathalie J. Bureau, Guy Cloutier

**Affiliations:** 1 Laboratory of Biorheology and Medical Ultrasonics, University of Montreal Hospital Research Center, Montreal, QC H2L 2W5 Canada; 2 Institute of Biomedical Engineering, University of Montreal, Montreal, QC H3C 3J7 Canada; 3 University of Sherbrooke, Sherbrooke, QC J1K 2R1 Canada; 4 University of Montreal Hospital, Montreal, QC H3X 0C1 Canada; 5 Department of Mathematics and Statistics, University of Montreal, Montreal, QC H2C 3J7 Canada; 6 Department of Neurology, University of Montreal, Montreal, QC H2C 3J7 Canada; 7 Department of Radiology, Radio-Oncology and Nuclear Medicine, University of Montreal, Montreal, QC H3C 3J7 Canada

**Keywords:** Ultrasound, Elastography, Biomechanics, Back muscles, Musculoskeletal conditions

## Abstract

**Objectives:**

To report the current elastography methods used to quantify back muscles’ biomechanical characteristics in patients with musculoskeletal disorders (MSKd) and inform on their reliability, validity, and responsiveness.

**Methods:**

MEDLINE, Embase, CINAHL, Cochrane library and grey literature were consulted. Predefined criteria allowed for study selection and data extraction. The quality of evidence was rated using the COSMIN tool. Data were meta-analyzed in terms of pooled intraclass correlation coefficient (pICC) for reliability and pooled standardized mean difference (pSMD) for validity and responsiveness. Heterogeneity was assessed.

**Results:**

Seventy-nine studies were included in the meta-analysis (total number of participants *N* = 3178). Three elastography methods were identified: strain imaging (SI; number of cohorts *M* = 26), shear wave imaging (SWI; *M* = 50), and vibration sonoelastography (VSE; *M* = 3). Strain imaging and SWI studies reported good reliability measurement properties (pICC > 0.70) and a medium pSMD (0.58 for SI and 0.60 for SWI; *p* ≤ 0.020) in discriminating MSKd from controls’ condition (validity). Strain imaging studies reported a medium pSMD (0.64; *p* = 0.005) in detecting within-group changes over time, whereas SWI pSMD was very high (1.24; *p* = 0.005). Only SWI reported significant but small pSMD (0.30; *p* = 0.003) in detecting between-group changes over time. The small number of VSE studies could not be meta-analyzed. Heterogeneity was high (I-squared > 90%; *p* < 0.001).

**Conclusions:**

Elastography presents good reliability results and a medium pSMD in discriminating MSKd from control conditions. Responsiveness data suggest detectable changes within groups over time using SI and SWI, calling for long-term longitudinal studies. Assessing changes between groups over time using elastography still needs to be proven. Highly significant heterogeneity limits meta-analytic results.

**Critical relevance statement:**

While still in its early-stage exploration phase, musculoskeletal ultrasound elastography may reliably quantify back muscles’ biomechanics in asymptomatic individuals, moderately discriminate back musculoskeletal disorders and detect biomechanical changes over time in these conditions, calling for long-term longitudinal studies.

**Key Points:**

- Ultrasound elastography is reviewed for back pain and related musculoskeletal disorder assessments.
- Growing literature supports good reproducibility, some validity and responsiveness.
- Back muscle elastography considers assumptions calling for standardized protocols.

**Graphical Abstract:**

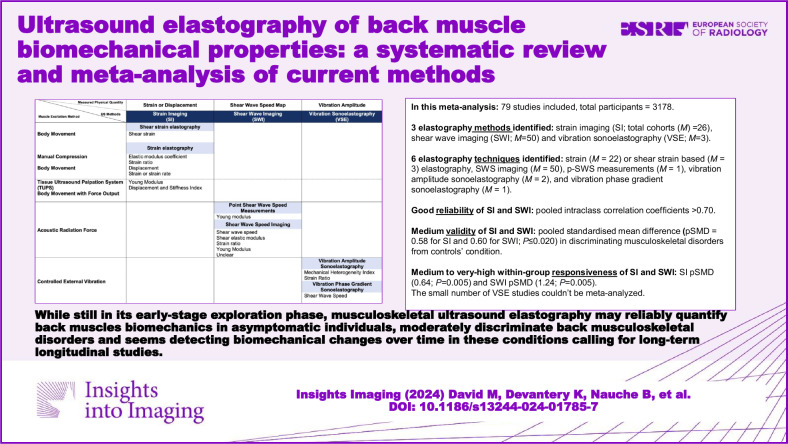

## Introduction

Musculoskeletal disorders (MSKd) are the most common cause of severe long-term pain and disability worldwide, responsible for 166 million (21.3%) years of life lived with disability in the general population [1]. In adults with MSKd, the most common pain is back pain, with neck and thoracic pain affecting up to 15% of older people, and low back pain (LBP) concerning up to 45% of the working-age population representing a major economic issue [1–4]. The interaction of forces and movement with the anatomy of back muscles, defined as biomechanics [5], is of prime importance for the comprehension of back pain [5–8]. Biomechanical models suggest that the activation of back muscles may depend on their inherent viscoelastic properties, which, placed under increased demand, may expose them to disorders and injuries [9–12]. Biomechanical properties such as viscoelasticity are influenced by the contraction or relaxation of the musculature [13], which makes them difficult to assess by conventional clinical imaging technologies such as computed tomography or magnetic resonance imaging. To answer this, the previous decades witnessed substantial research in the field of ultrasound imaging [14] with the aim of evaluating muscle and related soft tissue morphology and function [15]. In this regard, using ultrasound elastography (a group of techniques for objectively assessing tissue strain or stiffness [16]), researchers quantified muscle movements and deformations in various pathologies and anatomical structures [17–19], including back muscles [16, 20–22].

However, to date, there is no consensus on which elastography method or parameter is useful to assess back muscle biomechanical characteristics. Moreover, the role of elastography in measuring biomechanical characteristics of back muscles depends on the extent to which measurements are consistent and free from error, namely reliability. In addition, this also implies that accurate conclusions can be drawn about measurements to make predictions or diagnosis on biomechanical outcomes, discriminating among patients with and without the disorder or treatment; that is validity. Moreover, elastography must demonstrate the ability to detect changes over time, namely responsiveness [23, 24]. To allow clinicians to make decisions and research with accurate data and substantial indicators of back muscle biomechanical behavior in patients with MSKd, it is therefore necessary to report on the reliability, validity, and responsiveness of elastography.

Hence, the questions of this systematic review were:

- What are the current elastography methods and parameters used to quantify back muscles’ biomechanical properties in patients with MSKd and/or asymptomatic individuals?
- Are these measurements reproducible, valid, and responsive?

## Methods

### Search strategy

This systematic review was reported following the Preferred Reporting Items for Systematic Reviews and Meta-Analyses (PRISMA) [25] and the Methodological Expectations of Cochrane Intervention Reviews (MECIR) [26] recommendations. The protocol was registered in the PROSPERO database (#CRD42020186482).

The following databases were searched for relevant studies on April 22, 2020: MEDLINE (via Ovid, 1946 to April 21, 2020; via PubMed, on April 15, 2020); Embase (via Ovid, 1974 to April 21, 2020); Cochrane Database of Systematic Reviews (via Ovid, 2005 to April 17, 2020), Cochrane Central Register of Controlled Trials (via Ovid) and, CINAHL Complete from inception. Search strategies designed by a librarian (B.N.) used text words and relevant indexing to identify studies about ultrasound elastography and back muscles. The MEDLINE strategy (Supplementary Table S1) was peer-reviewed by a second librarian and then applied to all databases, with modifications to search terms as necessary. No language limits were applied. Case reports and animal studies were excluded. A grey literature search was conducted on February 10, 2021, to complement missing articles using the tool “Grey Matters: a practical tool for searching health-related grey literature” developed by the Canadian Agency for Drugs and Technologies in Health (CADTH) [27]. Further studies were identified by screening references of selected studies. The MEDLINE strategy was rerun on February 16, 2023 (2213 new citations were found).

### Study selection

Covidence systematic review software (Veritas Health Innovation, Melbourne, Australia) was used for data management. One reviewer (M.D.) screened titles and abstracts for eligibility according to criteria. Two independent reviewers (M.D. and K.D.) screened full texts for final inclusion. Inclusion criteria were: (1) a peer-reviewed full article, (2) focusing on back muscles or fascia as listed in the Terminologia Anatomica [28, 29], (3) in vivo measurements conducted on humans, (4) using ultrasound elastography, (5) recording biomechanical outcomes, (6) reporting some form of reliability, validity or responsiveness. Studies were considered assessing reliability if inter-rater, intra-rater or test-retest intraclass correlation coefficient (ICC) single measure (ICC Model 1, 2 or 3 form 1) were reported [24]. Studies reporting standard error of the measurement (SEM) or minimum detectable change (MDC) reliability scores were also included [30]. Studies were considered reporting validity if they were dealing with hypothesis testing for construct validity. “Hypothesis testing for construct validity” was defined as an ongoing process of learning more about ultrasound techniques used to quantify back muscles’ biomechanical properties, making new predictions, and testing them [23, 24, 31–33]. It could take the form of known-groups validation or convergent and discriminant validation. “Known-groups validation” was reported in terms of significant difference between measurements of extreme groups known to be different (i.e., MSKd versus controls). “Convergent or discriminant validation” reported how closely ultrasound scores are correlated to other variables linked to the disease (i.e., pain, worsening sensation, disease duration, disability, clinical diagnosis) or measures of the same construct to which it should be related (i.e., stiffness as measured with a muscle hardness meter). Finally, studies provided evidence of “responsiveness” if they measured changes over time (“within-group responsiveness”) or differences between groups after treatment (“between-groups responsiveness”) [23, 24, 31–33]. Exclusion criteria were: (1) no back muscle; (2) studies conducted only on cadavers, animals, tendons, or ligaments; (3) no elastography measurements; (4) no quantitative measurements; (5) no biomechanical outcome; (6) no evidence of reliability, validity, or responsiveness.

### Data extraction

Data were extracted from included studies by one reviewer (M.D.) and double-checked for accuracy by a second reviewer (K.D.). The following study characteristics were compiled: (1) authors’ details and demographics; (2) evidence of reliability, validity, and responsiveness; (3) elastography method, ultrasound probe, and biomechanical parameters; (4) anatomical structure, participant’s position, muscle/fascia state; and (5) clinical diagnosis.

Based on the World Federation for Ultrasound in Medicine and Biology (WFUMB) elastography classification [34], studies were sorted by (1) elastography method (i.e., strain imaging (SI), shear wave imaging (SWI), and vibration sonoelastography (VSE) [35]), (2) muscle excitation method (i.e., manual compression, body movement, tissue ultrasound palpation system (TUPS), acoustic radiation force, and controlled external vibration), (3) implemented elastography technique (i.e., strain elastography, shear strain elastography, point-shear wave speed (p-SWS), shear wave speed (SWS) imaging, vibration amplitude sonoelastography, and vibration phase gradient sonoelastography), and (4) elastography outcome (i.e., displacement, strain, shear strain, strain ratio, strain rate, SWS, shear modulus, shear elastic modulus, Young’s modulus, stiffness index, elastic modulus coefficient, and mechanical heterogeneity index).

### Data pooling and analysis

A multistage grouping of outcomes facilitated data pooling. First, we segregated data into reliability, validity, or responsiveness categories (some studies could serve multiple classifications). Second, using a random-effect model, ICCs (for reliability) and biomechanical outcomes (for validation and responsiveness) of studies assessing multiple muscles or conditions were pooled to have only one ICC (pooled ICC, pICC) or pooled standardized mean difference (pSMD; the standardized mean difference being defined as the ratio of the difference in means with the pooled standard deviation [36, 37]) and their respective 95% confidence interval (CI) by study. Pooled ICCs were based on estimates derived from Fisher transformation *z* = 0.5 ln ((1 + ICC) / (1 − ICC)), which has an approximate variance of Var(*z*) = 1 / (*N* − 3), where *N* is the study sample size (number of participants). Third, these pICC and pSMD by study were meta-analyzed to compute reliability, validity, and responsiveness pooled results. Note that reliability data were returned to their original metric for ease of interpretation of results [38]. As the meta-analysis was a priori set to assess only the ICCs and standardized mean difference, the number of studies included in the meta-analysis was maximized by estimating ICCs of articles reporting only the MDC or the SEM, following [30, 39]:

$${ICC}=1-\frac{{{SEM}}^{2}}{{{SD}}^{2}}$$

with SD the standard deviation and SEM being:

$${SEM}=\frac{{MDC}}{1.96\times \sqrt{2}}$$

Heterogeneity was assessed using Tau-squared, *Q* and *I*-squared statistics estimating the between-studies variance, the existence of true heterogeneity, and the percentage of the variability in effect estimates that it is due to heterogeneity, respectively [40]. *I*-squared percentages of 25%, 50%, and 75% were considered to report, respectively, low, medium, and high heterogeneity [40]. Heterogeneity statistics were calculated if there were sufficient studies included in the category of interest to perform a meta-regression analysis (rule of thumb of at least ten studies per variable). To look for potential variables predicting the variance in pSMD across studies, the meta-regression analysis was performed with a random-effects model using the restricted maximum likelihood estimation and applying the Knapp Hartung adjustment. The SPSS software (version 28.0.1.0) was used for statistical analysis. The significance level was set at *p* < 0.05.

### Quality of evidence grading

**Fig. 1:**
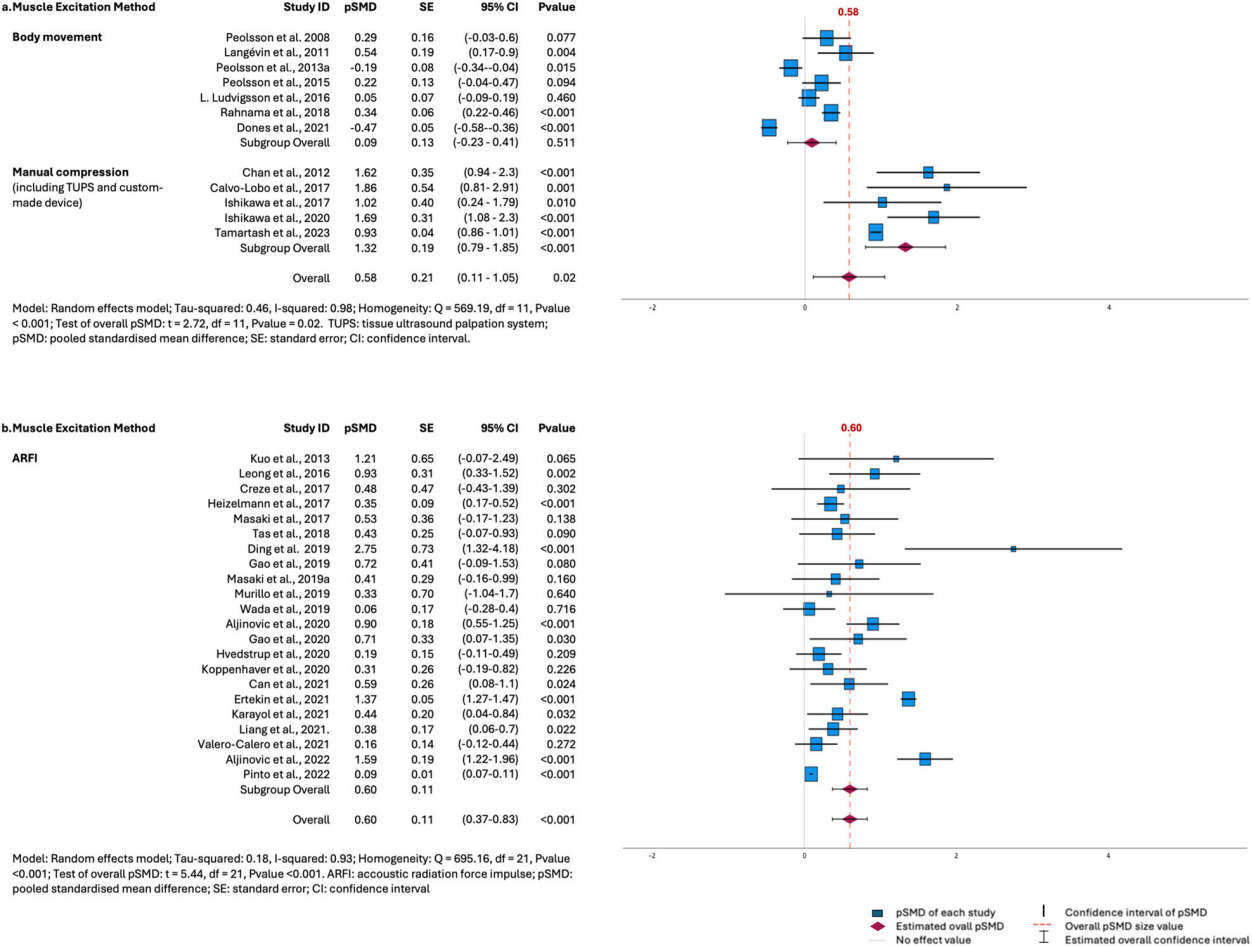
Forest plots of known-groups validation studies. **a** Known-groups validation studies using strain imaging. **b** Known-groups validation studies using shear wave imaging

As done before [39, 41], the quality of evidence of included studies was graded as high, moderate, low, and very low using the consensus-based standards for selecting health status measurement instruments (COSMIN) three-step methodology, initially dedicated to patients’ reported outcomes [31–33]. First, one reviewer (M.D.) used the COSMIN subscales for reliability, validity, and responsiveness to rate every single study as very good, adequate, doubtful, or inadequate (Supplementary Table S2). Twenty-six studies were rated by a second reviewer (K.D.) to validate the outcomes of the first reviewer (Cohen’s Kappa = 0.72). Disagreements were resolved by consensus or intercession of a third reviewer (G.C.). Second, pICCs and pSMD were rated against the criteria for good measurement properties as sufficient (pICC ≥ 0.70) or insufficient (pICC < 0.70) (for reliability) [31–33] and as very small (pSMD = 0.01), small (pSMD = 0.20), medium (pSMD = 0.50), large (pSMD = 0.80), very large (pSMD = 1.20), and huge (pSMD = 2.00) standardized mean difference (for validation and responsiveness) [36, 37, 42]. Rating was not determinate for unpooled ICC or standardized mean difference (categories including only one study). Third, a modified Grading of Recommendations Assessment, Development, and Evaluation (GRADE) approach was used to assign a final quality-evidence score that was downgraded when there was a risk of bias, imprecision (e.g., small study sample sizes) or inconsistency (appreciated by visual inspection of forest-plots (e.g., Fig. 1 and Supplementary Fig. S1)). Indirectness and publication bias were not considered in this modified GRADE approach, as the first was solved by exclusion criteria, and the second was discarded by the presence of natural heterogeneity of the populations included in the subgroups.

## Results

### Search results

**Fig. 2:**
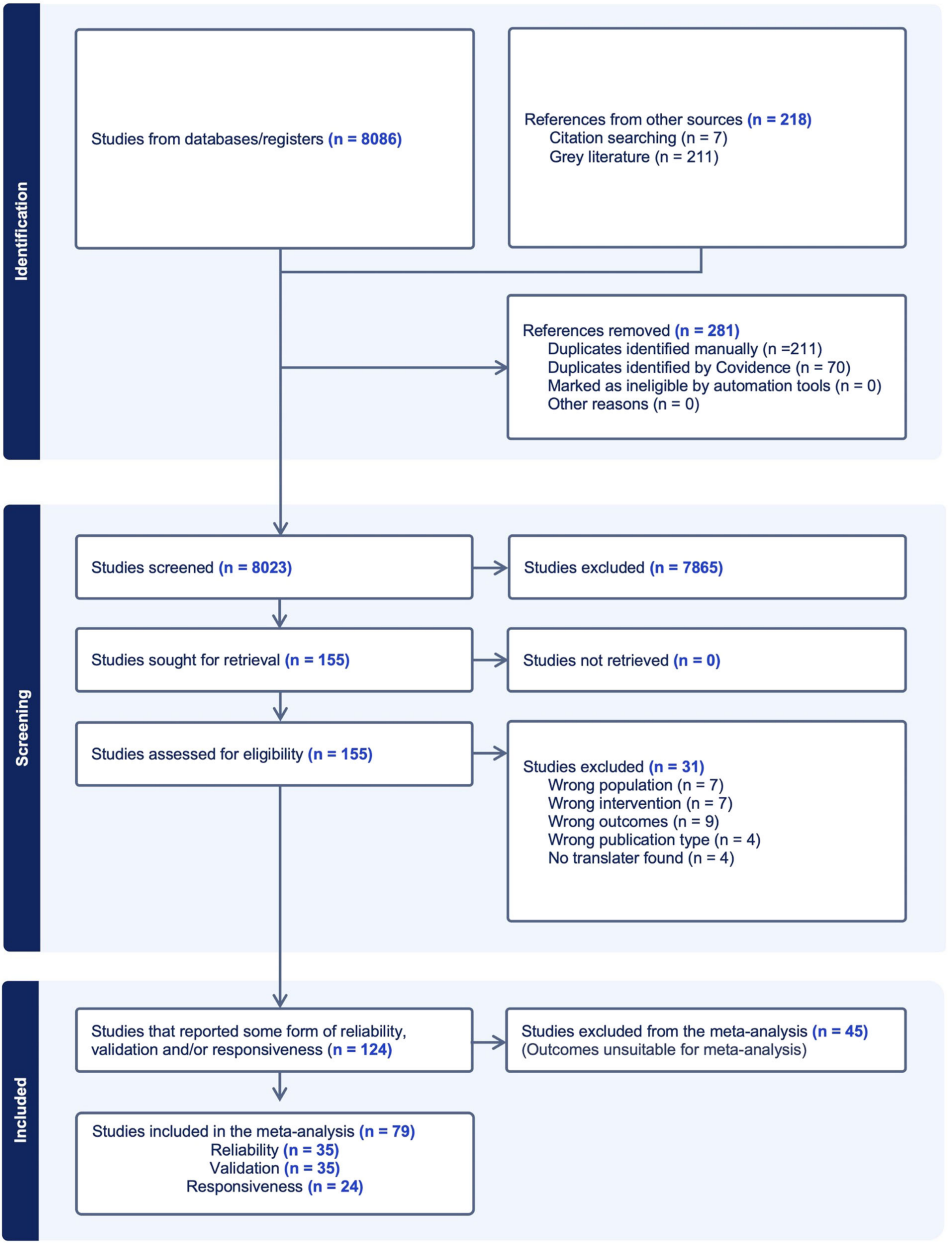
The Preferred Reporting Items for Systematic Reviews and Meta-Analyses (PRISMA) flow chart of the literature search. Studies may contribute to both reliability, validity, and responsiveness

Figure 2 presents the flow chart of the meta-analysis selection process. The initial database search identified 8086 records. A search in the grey literature yielded 211 additional articles. Seven papers were added from other sources. After removing duplicates, titles/abstracts, and full-text screening, we considered 124 studies reporting some form of reliability, validity, and responsiveness. From these studies, 79 presented sufficient consistency in data reporting to allow for data pooling and were included in the meta-analysis [43–121].

### Characteristics of studies included in the meta-analysis

#### Study design and demographics

Publications extended from 2008 to 2023. Fifty-seven studies [43–45, 48, 50–55, 57, 59, 61, 64, 65, 67, 68, 71–73, 75–97, 99–102, 104, 105, 111–116, 118, 121] were cross-sectional, nine were longitudinal, eight were randomized controlled trials (RCT), and five were non-RCTs. The total number of participants *N* was 3178, with an average of 40.2 participants per study (minimum = 6 [55], maximum = 278 [64]). The age was 36.9 (mean) ± 11.9 (SD). Women represented 27% of the population. See Supplementary Table S3 for a breakdown of study characteristics.

#### Diagnosis of included subjects

Thirty-one studies recruited asymptomatic participants, whereas patients with LBP (*M* = 19), neck or shoulder pain (*M* = 6), whiplash-associated disorders (*M* = 5), myofascial pain (*M* = 8), and rotator cuff tendinopathy (*M* = 3) were investigated. Among low back pain patients, studied groups presented unilateral lumbar disk herniation [43, 51], nonspecific lumbopelvic pain [50], and asymmetric lumbar somatic dysfunction [59, 60]. One cohort was also composed of medical workers [84] and another of middle-aged and elderly women [85]. Twelve studies did not provide any further clarification as to the type of LBP [49, 52, 72, 74, 79, 87, 95, 103, 104, 110, 111, 120]. Among neck and shoulder pain patients, studied groups presented rounded shoulders [63], migraine with and without ictal neck pain [65], neck and shoulder complaints [66], chronic neck pain [77, 105], and frozen shoulder in the freezing or frozen phase [112]. Two studies recruited subjects with cervical disc diseases. Each of the following conditions—knee flexion contracture, osteoporosis, fibromyalgia, and participants aged over 60 years—has been the subject of only one study [56, 64, 68, 117].

#### Patient position, muscle state and anatomical structure assessed

Most of the measurements were taken in seated (*M* = 36) and prone (*M* = 37) positions. In five studies, participants were upright. One study reported unclear position information [111]. Protocols were designed mostly with muscles at rest (*M* = 61) versus contracted (*M* = 16), stretched (*M* = 1) and passively mobilized (*M* = 3). In two studies, the muscle state was unclear [55, 111]. Thirty-nine studies evaluated intrinsic back muscles compared to 34 dedicated to extrinsic ones. Five studies assessed both categories. One study reported unclear information [83].

#### Applied elastography technology

**Fig. 3:**
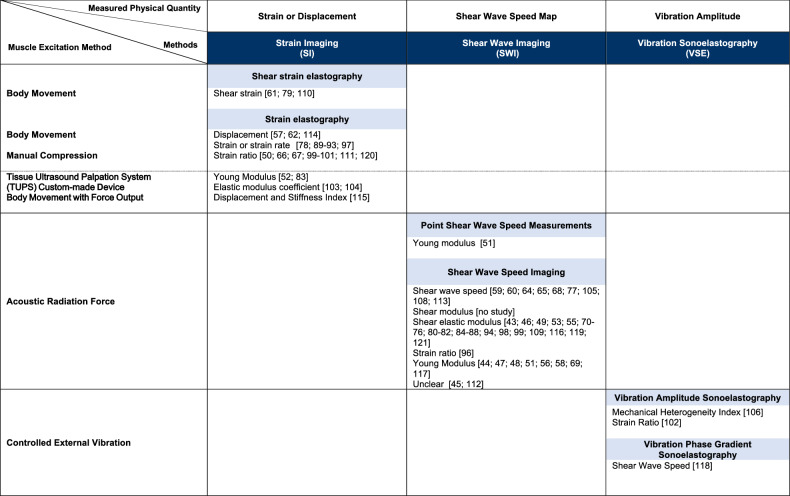
Applied elastography technologies classification inspired by Shiina et al [34]. Each column shows methods and measured physical quantities for elastography. Each row shows methods for inducing displacement. Each cell shows a type of elastography

Twenty-five papers reported the use of SI, 50 the use of SWI, and 3 the use of VSE (see Table 1 and Fig. 3 for definitions and classification, respectively). One manuscript used both SI and SWI [99] and was classified in the SI category for the statistical analysis. Among studies using SI, 12 applied manual compression, whereas 14 used body movement as the muscle excitation method. Amongst manual compression studies, four used a muscle excitation method with known applied stress (conversely to other manual compression studies for which the applied stress was unknown). Chan et al [52] and Ma et al [83] used a TUPS with an in-series force sensor included in the ultrasound probe to infer the Young modulus from the known applied stress. Tamartash et al [103, 104] built a custom-made system with a force gauge attached to the ultrasound transducer with the aim of inferring an elastic modulus coefficient from the stress/strain ratio. Amongst body movement excitation method studies, Wong et al [115] asked participants to perform a contraction of the latissimus dorsi while a load cell apparatus recorded the force output. Displacement and stiffness were inferred from the force and muscle-fascia junction displacement recordings. An acoustic radiation force is used in SWI, whereas a controlled external vibration excites the tissue in VSE. The acoustic radiation force produced by the focused beam of the ultrasound probe locally vibrates the tissue to produce propagating shear waves in SWI [34]. In VSE, controlled external vibrations are generated by an adapted external hand-held vibrating massager. We identified six elastography techniques: strain (number of cohorts or studies *M* = 22) or shear strain-based elastography (*M* = 3), SWS imaging (*M* = 50), p-SWS measurements (*M* = 1), vibration amplitude sonoelastography (*M* = 2), and vibration phase gradient sonoelastography (*M* = 1). For SI, strain ratio was the most reported outcome (*M* = 8). For SWI, the shear elastic modulus was to most reported outcome (*M* = 28). Essentially, with four exceptions [43, 48, 52, 54], all ultrasound probes were linear arrays. All were placed longitudinally to the muscle fibers’ direction. The most common frequencies used were within 2–10 MHz (*M* = 19). Fifty-five authors used a clinical scanner versus 24 that used a scanner equipped with a non-commercial postprocessing research software.

**Table 1:** Definitions of the elastography methods reported in the meta-analysis

Ultrasound method	Definitions inspired by [34] and [35]:
Strain imaging	Strain imaging calculates the Young’s modulus ($E$) or displays the distribution of strain components ($\varepsilon$) or normalized strain components within a region of interest after externally or internally applying a stress ($\sigma$): $E=\sigma /\varepsilon .$ Strain components include axial, lateral, and shear strain metrics or transformations of those metrics into a new mechanical descriptor.
Shear wave imaging	Under certain assumptions, shear wave imaging calculates $E$ or the shear modulus ($G$) after propagating shear waves and measuring their speed according to: $E=2\left(1+v\right)G=3G={3\rho {c}_{s}}^{2},$ where $v$ is the Poisson’s ratio, $\rho$ the tissue density and ${c}_{s}$ the shear wave speed. This modeling considers a purely elastic material (i.e., no viscosity). Shear waves are induced by an acoustic radiation pressure or an external vibration.
Vibration sonoelastography	Vibration sonoelastography calculates the disturbance in the amplitude of the vibration patterns within a tissue by externally applying a low-frequency vibration (20–1000 Hz) to induce internal vibrations. Doppler-based detection is used to determine the modulation parameter $\beta$ of a Bessel function that is proportional to the vibration amplitude of the vibrating target: $\beta =\,\sqrt{2\,}\left(\frac{{\sigma }_{\omega }}{{\omega }_{L}}\right),$ where ${\sigma }_{\omega }$ is the spectral spread of the vibration and ${\omega }_{L}$ the vibration frequency of the vibrating target. Mapping both the amplitude and the phase of the low-frequency wave propagation inside the tissue allows to derive the wave propagation velocity and its frequency dispersion related to the tissue viscosity.

#### Evidence of reliability, validity, and responsiveness

The final 79 papers included in the meta-analysis contain 52 reliability coefficients (reported in 35 studies), 35 known-groups validation outcomes (participants with MSKd versus controls), 21 within-group responsiveness outcomes, and 8 between-groups responsiveness outcomes, as a single study may contribute to more than one category (Supplementary Table S4 presents pooled results by study). Due to inconsistent data reporting, we could not meta-analyze known-groups validation outcomes from groups varying in experimental settings (trunk posture, muscular strength) or muscles assessed. The same applies to convergent and discriminant validation outcomes that were too inconsistent to be meta-analyzed.

### Pooled ICCs for reliability studies

Table 2 presents pICCs obtained for the different elastography methods. For SI, data from two test-retest, seven intra-rater and two inter-rater reliability studies accounting for moderate, high and low quality of evidence, respectively, suggested sufficient criteria for good measurement properties (test-retest pICC (95% CI) = 0.93 (0.42–0.99), *M* = 2, *N* = 87; intra-rater pICC (95% CI) = 0.85 (0.70–0.92), *M* = 7, *N* = 226; inter-rater pICC (95% CI) = 0.79 (0.67–0.87), *M* = 2, *N* = 36). The SI ICCs were gathered only from data of asymptomatic participants if one subtracted a study reporting unclear information [79]. For SWI, data from 8 test-retest, 20 intra-rater and 10 inter-rater reliability studies accounting for high quality of evidence suggested sufficient criteria for good measurement properties (test-retest pICC (95% CI) = 0.87 (0.73–0.94), *M* = 8, *N* = 149; intra-rater pICC (95% CI) = 0.87 (0.82–0.92), *M* = 20, *N* = 522; inter-rater pICC (95% CI) = 0.88 (0.82–0.92), *M* = 10, *N* = 204). Although SWI ICCs were mostly gathered from data of asymptomatic participants, noted however three exceptions where ICCs were gathered from participants with a unilateral disk herniation [43], a whiplash-associated disorder [46], and myofascial pain [99]. Noted also two studies providing unclear information on this aspect [44, 60]. For VSE, the rating was not determinate as data were coming from only one test-retest study [106] and one intra and inter-rater reliability study [118].

**Table 2:** Summary of findings for reliability studies

Strain imaging			
	T-R	INTRA-R	INTER-R
Pooled ICC result^a^	0.93 (0.42–0.99) *M* = 2 *N* = 87	0.85 (0.70–0.92) *M* = 7 *N* = 226	0.79 (0.67–0.87) *M* = 2 *N* = 36
Overall rating^b^	+	+	+
Quality of evidence^c^	Moderate	High	Low
Studies included	[78, 89]	[52, 57, 79, 83, 93, 100, 101]	[83, 101]

Shear wave elastography			
	T-R	INTRA-R	INTER-R
Pooled ICC result^a^	0.87 (0.73–0.94) *M* = 8 *N* = 149	0.87 (0.82–0.92) *M* = 20 *N* = 522	0.88 (0.82–0.92) *M* = 10 *N* = 204
Overall rating^b^	+	+	+
Quality of evidence^c^	High	High	High
Studies included	[55, 73, 75, 81, 96, 99, 116, 121]	[44, 46, 48, 53, 59, 69, 71, 73, 75, 81, 86–88, 94, 96, 98, 99, 113, 116, 119]	[43, 46, 59, 75, 76, 81, 86, 113, 119, 121]

Vibration sonoelastography			
	T-R	INTRA-R	INTER-R
Pooled ICC result^a^	0.98 (0.96–0.99) *M* = 1 *N* = 48	0.91 (0.76–0.96) *M* = 1 *N* = 23	0.83 (0.56–0.94) *M* = 1 *N* = 48
Overall rating^b^	?	?	?
Quality of evidence^c^	Very low	Low	Low
Studies included	[106]	[118]	[118]

*ICC* intraclass correlation coefficient, *T-R* test-retest reliability, *INTRA-R* intra-rater reliability, *INTER-R* inter-rater reliability, *M* number of cohorts, *N* number of participants

^a^ Pooled intraclass correlation coefficient (pICC) results based on estimates derived from a Fisher transformation using a random-effect model and their related 95% confidence interval

^b^ Overall rating was graded as sufficient “+” (pICC ≥ 0.70) or insufficient “–” (pICC < 0.70). Categories with a single record were not rated and noted as “?”

^c^ Quality of evidence (i.e., high, moderate, low, very low) graded using a modified Grading of Recommendations Assessment, Development, and Evaluation (GRADE) approach

### Pooled standardized mean differences

Results on pSMD are presented in Table 3 and discussed below.

**Table 3:** Summary of findings for validation and responsiveness studies

Strain imaging			
	KG	WG	BG
pSMD result^a^	0.58 (0.11–1.05) SE = 0.21 *M* = 12 *N* = 569	0.64 (0.25–1.02) SE = 0.17 *M* = 9 *N* = 302	0.19 (−0.36 to 0.73) SE = 0.13 *M* = 3 *N* = 163
Significancy^a^	*p* = 0.020	*p* = 0.005	*p* = 0.278
Size of the pSMD^b^	Medium	Medium	Very small
Quality of evidence^c^	High	High	Low
Included studies	[50, 52, 57, 66, 67, 78, 79, 90–92, 97, 104]	[61, 62, 92, 99, 103, 110, 111, 115, 120]	[61, 99, 114]

Shear wave imaging			
	KG	WG	BG
pSMD result^a^	0.60 (0.37–0.83) SE = 0.11 *M* = 22 *N* = 1578	1.24 (0.46–2.02) SE = 0.35 *M* = 12 *N* = 390	0.30 (0.17–0.43) SE = 0.05 *M* = 5 *N* = 363
Significancy^a^	*p* < 0.001	*p* = 0.005	*p* = 0.003
Size of the pSMD^b^	Medium	Very large	Small
Quality of evidence^c^	High	High	High
Included studies	[44, 45, 51, 54, 56, 58–60, 64, 65, 68, 72, 77, 80, 82, 84, 85, 87, 95, 105, 108, 112]	[47, 49, 56, 59, 63, 70, 76, 98, 107, 109, 117, 119]	[45, 49, 63, 74, 107]

Vibration sonoelastography			
	KG	WG	BG
pSMD result^a^	1.96 (−2.43 to 6.34) SE = 1.02 *M* = 1 *N* = 50	-	-
Significancy^a^	*p* = 0.055	-	-
Size of the pSMD^b^	Indeterminate	-	-
Quality of evidence^c^	Moderate	-	-
Included studies	[102]		

*KG* known-group validation, *WG* within-group responsiveness, *BG* between-groups responsiveness, *pSMD* pooled standardized mean difference, *SE* standard error, *M* number of cohorts, *N* number of participants, *p* *p* value associated with the standardized mean difference

^a^ Pooled standardized mean difference (pSMD) results obtained using a random-effect model, and their related 95% confidence interval

^b^ The size of the pooled standardized mean difference (pSMD) was graded as 0.01 (very small), 0.2 (small), 0.5 (medium), 0.8 (large), 1.2 (very large), 2.0 (huge) according to ref. [37]

^c^ The quality of evidence (i.e., high, moderate, low, very low) graded using a modified Grading of Recommendations Assessment, Development, and Evaluation (GRADE) approach

#### Pooled standardized mean differences for known-groups validation studies

Data from twelve studies accounting together for high quality of evidence suggested a medium and significant (*p* = 0.020) pSMD for SI in discriminating between patients with MSKd and controls (pSMD = 0.58 (95% CI = 0.11–1.05), *M* = 12, *N* = 569) [50, 52, 57, 66, 67, 78, 79, 90, 91, 92, 97, 104]. Data from 22 studies accounting for high quality of evidence suggested a medium and significant (*p* < 0.001) pSMD for SWI in discriminating between patients with MSKd and controls (pSMD = 0.60 (95% CI = 0.37–0.83), *M* = 22, *N* = 1578) [44, 45, 51, 54, 56, 58–60, 64, 65, 68, 72, 77, 80, 82, 84, 85, 87, 95, 105, 108, 112]. For VSE, pSMD rating was not determinate as there was only one single study in this category [102].

#### Pooled standardized mean differences for within-group responsiveness studies

Data from nine studies accounting together for high quality of evidence suggested a medium and significant (*p* = 0.005) pSMD for SI in detecting changes in biomechanical properties of muscles over time (pSMD = 0.64 (95% CI = 0.25–1.02), *M* = 9, *N* = 302) [61, 62, 92, 99, 103, 110, 111, 115, 120]. Data from twelve studies accounting together for high quality of evidence suggested a very large and significant (*p* = 0.005) pSMD for SWI in detecting changes in biomechanical properties of muscles over time (pSMD = 1.24 (95% CI = 0.46–2.02), *M* = 12, *N* = 390) [47, 49, 56, 59, 63, 70, 76, 98, 107, 109, 117, 119].

#### Pooled standardized mean differences for between-groups responsiveness studies

Data from three studies accounting together for low quality of evidence suggested a very small and non-significant (*p* = 0.278) pSMD for SI in detecting changes in biomechanical properties of muscles between groups after treatment (pSMD = 0.19 (95% CI = −0.36 to 0.73), *M* = 3, *N* = 163) [61, 99, 114]. Data from five studies accounting together for high quality of evidence suggested a small and significant (*p* = 0.003) pSMD for SWI in detecting changes in biomechanical properties of muscles between groups after treatment (pSMD = 0.30 (95% CI = 0.17–0.43), *M* = 5, *N* = 363) [45, 49, 63, 74, 107].

### Heterogeneity and meta-regression

Heterogeneity statistics were performed on SI and SWI studies reporting known-groups validation and within-group responsiveness outcomes. The *Q*-statistic value was significant for each category (*p* < 0.001). Tau-squared and *I*-squared values ranged between 0.182–1.219 and 90–99.3%, respectively (Table 4). There was a significant and large amount of heterogeneity within the selected dataset. To explain this variance in pSMD, we tested several predictors using univariate meta-regression (Supplementary Table S5). The analysis indicated that the excitation method (body movement versus manual compression) significantly explained 76% of the variance among pSMD for SI studies discriminating between patients with MSKd and controls (*p* < 0.001) (Table 5 and Fig. 1). No other significant predictor was found in any category, nor for SWI.

**Table 4:** Results of heterogeneity analysis

Known-groups validation	Heterogeneity statistics		Chi-square (*Q* statistic)	*M*, *N*	Sig.
SI	Tau-squared	0.463	569.191	12, 569	*p* < 0.001
	*I*-squared (%)	98.3			
SWI	Tau-squared	0.182	695.162	22, 1578	*p* < 0.001
	*I*-squared (%)	93.2			

Within-group responsiveness	Heterogeneity statistics		Chi-square (*Q* statistic)	*M*, *N*	Sig.
SI	Tau-squared	0.206	109.884	9, 302	*p* < 0.001
	*I*-squared (%)	90.0			
SWI	Tau-squared	1.219	605.118	12, 390	*p* < 0.001
	*I*-squared (%)	99.3			

Between-groups responsiveness	Heterogeneity statistics		Chi-square (*Q* statistic)	*M*, *N*	Sig.
SI	Tau-squared	*M* = 3, number of records is insufficient for heterogeneity statistics.			
	*I*-squared (%)				
SWI	Tau-squared	*M* = 5, number of records is insufficient for heterogeneity statistics.			
	*I*-squared (%)				

*SI* strain imaging, *SWI* shear wave imaging, *Tau-squared* variance of the standardized mean difference across studies, *I-squared* proportion of total variance between studies that is attributed to heterogeneity, *Q* heterogeneity statistic, *M* number of cohorts, *N* number of participants, *Sig.* *p* value associated with the *Q* statistic

**Table 5:** Parameter estimates for known-groups validation studies assessing strain imaging outcomes

Parameters	Estimate	S.E.	*t*	Sig. (2-tailed)	95% confidence interval		Univariate meta-regression
					Lower	Upper	
**US excitation method:**							*p* < 0.001, *R*^*2*^ = 76 *M* = 12, *N* = 558
(Intercept)	1.314	0.1947	6.746	*p* < 0.001	0.880	1.747	
Body movement	−1.222	0.2328	−5.251	*p* < 0.001	−1.741	−0.704	
Manual compression	0^a^						

*US* ultrasound, *SE* standard error, *t* *t* statistic, *M* number of cohorts, *N* number of participants, *Sig.* *p* value associated with the *t* statistic, *R*^*2*^ proportion of total variance that is explained by the ultrasound excitation method used

^a^ This parameter is set to zero because it is redundant

## Discussion

This systematic review included 79 studies reporting the use of strain imaging (SI), shear wave imaging (SWI) and vibration sonoelastography (VSE) to assess the biomechanical properties of back muscles in MSKd. Whereas the small number of VSE studies could not be meta-analyzed, SI and SWI studies demonstrated good reliability results, moderate validity to discriminate between patients with MSKd and controls, and moderate to very high within-group responsiveness, for SI and SWI, respectively. Strain imaging and SWI between-groups responsiveness is more questionable, partly due to the lack of sufficient studies available.

Reliability considers the sum of measurement errors and patient variability, that is, the interaction between the tool used and the population of interest [24]. Given its context-dependency, reliability is essential to be evaluated before any testing process. In this meta-analysis, we summarized the reliability characteristics of elastography to estimate back muscle biomechanics in participants with MSKd or, where necessary, asymptomatic participants. However, notice that the reliability coefficients of studies included in our review were all but three [43, 46, 99] calculated on young (31.1 ± 12.5 years) asymptomatic participants. Therefore, it is questionable whether the reliability characteristics demonstrated here apply to the MSKd population.

Both test-retest, intra and inter-rater reliability of SI, SWI and VSE studies presented sufficient criteria for good measurement properties (pICC > 0.70). Strain imaging studies demonstrated slightly higher values than SWI studies for test-retest reliability (pICC (95% CI) = 0.93 (0.42–0.99) for SI versus 0.87 (0.73–0.94) for SWI). Both SI and SWI indicated good inter and intra-rater reliability with pICC (95% CI) ranging from 0.79 (0.67–0.87) to 0.88 (0.82–0.92). Note, however, that most SI evidence comes from moderate (test-retest) to low-quality (inter-rater) evidence contrary to high-quality SWI evidence. The discrepancy in the quality of evidence between SI and SWI studies may be due to insufficient details on the SI data collection or analysis procedure to permit replication. By contrast, intra-rater reliability procedures in SI studies were more documented, resulting in a higher quality of evidence index.

Forty-three of the 52 reliability coefficients contained in the meta-analysis (83%) were acquired at rest. Interestingly, four studies also reported the reliability of SI during active movements in trapezius, neck muscles and lumbar multifidus [52, 57, 78, 89], allowing investigation of soft-tissues torque-dependent biomechanical changes in MSKd physiopathology [122]. Altogether, moderate to excellent reliability was reported (minimum pICC (95% CI) = 0.61 (0.45–0.90); maximum pICC (95% CI) = 0.99 (0.98–0.99)). Five SWI studies also reported moderate to excellent reliability coefficients (minimum pICC (95% CI) = 0.73 (0.59–0.81); maximum pICC (95% CI) = 0.99 (0.98–1.00)) during muscle contraction on the trapezius [75, 81, 98, 121] and lumbar multifidus [73]. While one can expect fewer measurement errors in SWI acquisitions (performed in isometric conditions) compared to SI (performed during concentric movements, except for TUPS), it is instructive to note that both SWI and SI reliability values were in the same range.

Strain imaging and SWI significantly (*p* ≤ 0.020) discriminate between participants with MSKd and controls. Both presented high-quality evidence and a medium pSMD. In the particular case of two normal distributions with the same variance, a pSMD of 0.01 (very small), 0.2 (small), 0.5 (medium), 0.8 (large), 1.2 (very large), 2.0 (huge) means that 0%, 14.7%, 33%, 47.4%, 62.2% and 81.1%, respectively, of the area covered by the two distributions is not overlapping [37].

Therefore, elastography allows discriminating between MSKd and controls in 33% of cases, the remaining 67% being subject to false positives or negatives. Increasing the discriminatory power could be done by enhancing the sample size or reducing variability. Knowing that the prevalence of MSKd in the general population is 21.3% [1], to discriminate among people with MSKd and controls with a power equal to 0.80 and *α* = 0.05, 254 participants should be recruited to meet these specifications [123]. The average known-groups validation study sample size in this review was 63 ± 55, which suggests insufficient numbers of participants to demonstrate discrimination among populations. On the other hand, heterogeneity statistics confirmed variability among studies. While the meta-regression has shown no evidence of variable influencing the variance among SWI studies, forest plot visual inspection (Fig. 1b) suggests a reduction in the magnitude of the confidence intervals as studies become more recent, while variance among studies does not seem to be improved. The reason for this could be a natural heterogeneity between studied groups (limiting meta-analytic results) combined with a possible improvement in protocols over time. Strain imaging meta-regression results pointed out a 76% contribution of the excitation method (body movement versus manual compression) to predict the variance among studies (Table 5). If SI studies are dichotomized according to their excitation method (Fig. 1a), the pSMD of studies using body movement as the excitation method was very small (0.09) and non-significant (*p* = 0.51). On the contrary, the pSMD of manual compression studies was very large (1.32) and significant (*p* < 0.001). Strain imaging using manual compression, cardiovascular pulsation or respiratory motion has successfully been used to characterize breast and prostate cancers [124], carotid plaques [125], or diaphragmatic breathing [126], respectively. However, using body movement to characterize large muscle structures may be a source of variability as it depends on the individual anatomy and force-sharing strategies, movement control, and muscle complex biomechanical behavior. Or the reason may lie elsewhere, as SI exhibits good reliability (pICC > 0.79). The hypothesis used to classify data by groups to calculate pSMD also deserves reflection. We dichotomized studies’ results as “data of muscles assumed stiffer/presenting less displacement” versus “data of muscles assumed less stiff/presenting more displacement” according to the hypothesis made by the authors or, if necessary, according to the state of the art. However, many authors did not disclose assumptions. Moreover, the original hypothesis underpinning that MSKd patients present stiffer soft tissues with less displacement due to pathophysiological processes (e.g., fibrosis, fatty infiltration, inflammation, or adhesions) [127], could perhaps be a specific case from the relatively wide range of assumptions applicable to all cases. For example, Dones et al [57] and Peolsson et al [91] reported conclusions against the state of the art, resulting in negative pooled ES disadvantaging the overall measure of pSMD in SI.

Strain imaging as well as SWI significantly (*p* < 0.005) detected changes within groups over time with high-quality evidence, reporting medium (0.64) and very large (1.24) pSMD, respectively. Therefore, SI can detect changes over time within subjects in 33% of cases versus 62.2% for SWI, the remaining 67% and 37.8%, respectively, being subject to false positives or negatives. As data were heterogeneous (Table 4), we performed a meta-regression to consider the delay between treatment and measurements influencing variability. Although the analysis was inconclusive (Supplementary Table S5), it is tempting to point out the relatively limited time between the treatment and ultrasound measurements, showing little regard for the chronicity of MSKd to identify changes in biomechanical outcomes. Indeed, aside from Sakaki et al [98] assessing changes in trapezius characteristics 1 year after arthroscopic rotator cuff surgery, measurements were made within 2 weeks [56], 72 h [99], 48 h [47], 24 h [119] or immediately after one-session treatment [49, 59, 61, 62, 76, 92, 107, 109, 111, 115]. Although some authors also measured biomechanical parameters at baseline and after 2 months [110], 4 weeks [63, 117], 3 weeks [120], and 2 weeks [103] treatments, long-term longitudinal studies are lacking and required. The same is true for between-groups responsiveness studies where only SWI presented, even if small (0.30) but significant (*p* = 0.003) pSMD with high-quality evidence (changes detected between groups over time in 14.7% of cases, the remaining 85.3% being subject to false positives or negatives). Aljinovic et al [45] found no difference in SWI values between recovered and non-recovered participants with whiplash injuries at 6 months of follow-up. They suggested a possible increase in adherence to physical medicine interventions post-trauma to distort the short-term biomechanical data collected. Koppenhaver et al [74] found significant differences in the erector spinae shear elasticity modulus (but failed for the multifidus) between groups 1 week after receiving dry needling (DN) or sham DN providing arguments that elasticity parameters should be studied in long-lasting studies conducted in the same context as clinical care, combined with other treatments. Moreover, as biomechanical characteristics may vary according to the muscle typology, designating the more sensitive structure to detect changes over time throughout the disease may be required [122].

### Limitations

Sixteen studies were excluded from the meta-analysis because an inaccurate ICC model or form was reported. This reduced the number of available evidence and weakened reported results. More, 18 reliability studies included in the meta-analysis mentioned no model or form details and were assumed to report a one-way mixed-effects ICC model for single measurements. Quality of evidence ratings were double-checked for only 1/3 of the papers by another reviewer. Due to limited resources, the screening of titles and abstract was performed by only one reviewer (however, two reviewers did the data extraction). The small number of responsiveness data reduced the chance of finding potential predictors for within-group responsiveness variability and did not allow to perform meta-regression for between-groups responsiveness. The body mass index was reported in only half of the studies, distorting predictor analysis for this variable. Finally, the highly significant heterogeneity between primary studies may limit meta-analytic results.

## Conclusions

This meta-analysis reported three elastography methods used to quantify back muscles’ biomechanical properties: SI, SWI and VSE. Despite good reliability results, muscular elastography is still in an early-stage exploration phase, partly able to discriminate between patients with MSKd and controls in cross-sectional studies. The type of methods used to excite the tissue may be a variability factor. Strain imaging and SWI seem to detect changes within groups over time, but this needs to be confirmed by long-term longitudinal studies. Assessing changes between groups over time using elastography still needs to be proven.

### Supplementary information

ELECTRONIC SUPPLEMENTARY MATERIAL

## Data Availability

Data analyzed are available upon request by contacting the corresponding author.

## Abbreviations

CI: Confidence interval
COSMIN: Consensus-based standards for the selection of health status measurement instruments
DN: Dry needling
GRADE: Grading of Recommendations Assessment, Development and Evaluation
ICC: Intraclass correlation coefficient
LBP: Low back pain
M: Number of cohorts or studies
MDC: Minimum detectable change
MSKd: Musculoskeletal disorders
N: Number of participants
pICC: Pooled ICC
pSMD: Pooled standardized mean difference
p-SWS: Point-shear wave speed measurements
RCT: Randomized controlled trials
SD: Standard deviation
SEM: Standard error of the measurement
SI: Strain imaging
SWI: Shear wave imaging
SWS: Shear wave speed
TUPS: Tissue ultrasound palpation system
VSE: Vibration sonoelastography
