# Supplementary material for: Ultrasound elastography of back muscle biomechanical properties: a systematic review and meta-analysis of current methods
Source: Insights Imaging. 2024 Aug 14;15:206. doi: 10.1186/s13244-024-01785-7 (PMC11324641; doi:10.1186/s13244-024-01785-7)
Supplement: Supplementary file 1 — ELECTRONIC SUPPLEMENTARY MATERIAL [file 13244_2024_1785_MOESM1_ESM.pdf]

Ultrasound elastography of back muscle biomechanical properties: a systematic review and meta-analysis of current methods

ELECTRONIC SUPPLEMENTARY MATERIAL

a.Muscle Excitation Method	Study ID	pSMD	SE	95% CI	Pvalue
**Body movement**	Peolsson et al., 2008	0.68	0.44	(-0.18-1.54)	0.121
	Wong et al., 2016	0.19	0.28	(-0.35-0.74)	0.489
	Griefhan et al., 2017	1.11	0.38	(0.37-1.85)	0.003
	Griefhan et al., 2021	0.75	0.29	(0.18-1.33)	0.010
	Vining et al., 2022	0.09	0.26	(-0.41-0.59)	0.724
	Subgroup overall	0.51	0.19	(-0.02-1.03)	0.055
**Manual compression**	Yurdakul et al., 2019	-0.10	0.10	(-0.3-0.1)	0.320
	Sanchez-Infante et al., 2021	1.04	0.06	(0.93-1.16)	<0.001
	Tamartash et al. 2022	0.97	0.10	(0.78-1.16)	<0.001
	Wachi et al., 2022	1.21	0.38	(0.47-1.96)	0.001
	Subgroup overall	0.75	0.30	(-0.21-1.71)	0.088
	Overall	0.64	0.17	(0.25-1.02)	0.005

Model: Random effects model; Tau-squared: 0.21, I-squared: 0.90; Homogeneity: Q = 109.88, df = 8, Pvalue = 0.005; Test of overall pSMD: t = 3.83, df = 8, Pvalue < 0.001. pSMD: pooled standardised mean difference; SE: standard error; CI: confidence interval.

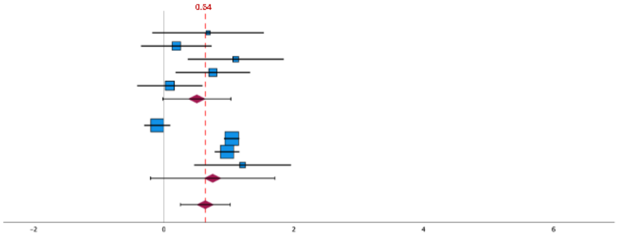

b.Muscle Excitation Method	Study ID	pSMD	SE	95% CI	Pvalue
**ARFI**	Xu et al., 2018	3.00	0.55	(1.92-4.09)	<0.001
	Ding et al., 2019	2.08	0.53	(1.04-3.12)	<0.001
	Gao et al., 2020	0.86	0.33	(0.21-1.51)	0.009
	Kisilewicz et al., 2020	4.53	0.71	(3.13-5.92)	<0.001
	Bethers et al. 2021	0.47	0.09	(0.29-0.64)	<0.001
	Buran Cirak et al. 2021	1.72	0.06	(1.61-1.83)	<0.001
	Kumamoto et al., 2021	1.37	0.11	(1.16-1.57)	<0.001
	Yamaura et al., 2021	0.81	0.07	(0.68-0.94)	<0.001
	Gunaydin et al., 2022	0.37	0.07	(0.24-0.5)	<0.001
	Sasaki et al., 2022	0.86	0.28	(0.3-1.42)	0.002
	Valero-Calero et al., 2022	-0.02	0.07	(-0.15-0.12)	0.800
	Vatovec et al., 2022	0.22	0.07	(0.09-0.36)	0.001
	Subgroup overall	1.24	0.35	(0.46-2.02)	0.005
	Overall	1.24	0.35	(0.46-2.02)	0.005

Model: Random effects model; Tau-squared: 1.22, I-squared: 0.99; Homogeneity: Q = 605.12, df = 11, Pvalue = 0.005; Test of overall pSMD: t = 3.50, df = 11, Pvalue <0.001. ARFI: accoustic radiation force impulse; pSMD: pooled standardised mean difference; SE: standard error; CI: confidence interval

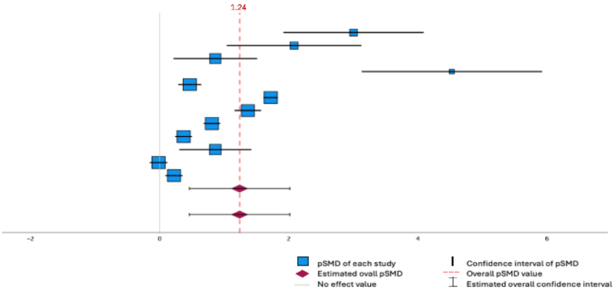

Figure S1.1 – **Forest Plots of responsiveness studies. a.** Within-group responsiveness studies using strain imaging. **b.** Within-group responsiveness studies using shear wave imaging.

a.Muscle Excitation Method	Study ID	pSMD	SE	95% CI	Pvalue
**Body movement**	Griefhan et al. 2021	0.39	0.18	(0.04-0.75)	0.031
	Weber et al., 2022	0.02	0.04	(-0.06-0.1)	0.657
	Subgroup overall	0.16	0.18	(-2.17-2.5)	0.536
**Manual compression**	Sanchez-Infante et al., 2021	0.34	0.27	(-0.19-0.87)	0.206
	Subgroup overall	0.34	0.27	(-0.19-0.87)	0.206
	Overall	0.19	0.13	(-0.36-0.73)	0.278

Test of overall pSMD: t = 1.47, df = 2, Pvalue = 0.28. pSMD: pooled standardised mean difference; SE: standard error; CI: confidence interval.

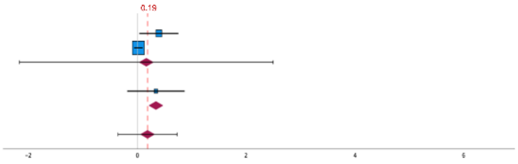

b.Muscle Excitation Method	Study ID	pSMD	SE	95% CI	Pvalue
**ARFI**	Buran Cirak et al. 2021	0.66	0.38	(-0.09-1.41)	0.086
	Alijnovic et al., 2022	0.11	0.15	(-0.19-0.41)	0.471
	Gunaydin et al., 2022	0.27	0.12	(0.04-0.49)	0.022
	Koppenhaver et al., 2022	0.35	0.06	(0.23-0.47)	<0.001
	Valero-Calero et al., 2022	0.18	0.21	(-0.23-0.58)	0.392
	Subgroup overall	0.30	0.05	(-0.23-0.58)	0.392
	Overall	0.30	0.05	(0.17-0.43)	0.003

Test of overall pSMD: t = 6.38, df = 4, Pvalue < 0.01. ARFI: accoustic radiation force impulse; pSMD: pooled standardised mean difference; SE: standard error; CI: confidence interval

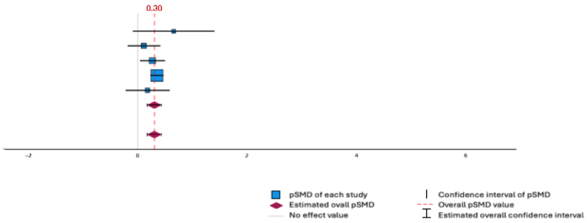

Fig S1.2 – **Forest Plots of responsiveness studies. a.** Between-groups responsiveness studies using strain imaging. **b.** Between-groups responsiveness studies using shear wave imaging.

Table S1 – Medline strategy. Ovid MEDLINE(R) ALL 1946 to February 15, 2023

1	exp back muscles/ or exp fascia/	13150
2	((back or dorsi or dorsal or vertebr* or neck or cervic* or nucha* or spina* or paraspinal* or sacrospinal* or thoracic* or thoracolumbar* or lumbar* or lumbor* or lumbosacral* or sacrococcygeal* or sacral* or coccygeal* or trapez* or levator scapulae* or rhomboid* or serratus posterior* or splenius or illiocostal* or longissimus or rotator* or multifidi* or semispinal* or interspinal* or intertransvers* or transversospinal*) adj8 (muscl* or muscul* or neuromusc* or fascia* or aponeuros*)).tw,kf.	64718
3	1 or 2	74897
4	exp ultrasonography/	482831
5	(ultrasonograph* or ultrasound* or ultrasonic* or sonograph* or sonogram* or echograph* or echogram* or echotomograph* or elastogra* or sonoelastogra* or ARFI or ((elasticity or visco* or deformation or supersonic or Nakagami or Homodyned) adj4 imaging) or speckle or Backscatter or attenuation or "speed of sound" or "shear wave*").tw,kf.	595784
6	4 or 5	828118
7	3 and 6	3772
8	case reports.pt.	2318689
9	(case* adj2 (report* or stud*)).ti.	422378
10	(case* adj2 report*).jw.	125837
11	8 or 9 or 10	2483057
12	7 not 11	3088
13	(Animals/ or Models, animal/ or Disease models, animal/) not Humans/	5059973
14	((animal or animals or canine* or cat or cats or dog or dogs or feline or hamster* or lamb or lambs or mice or monkey or monkeys or mouse or murine or pig or pigs or piglet* or porcine or primate* or rabbit* or rats or rat or rodent* or sheep* or veterinar*) not (human* or patient* or women or men)).ti,kf,jw.	2566909
15	13 or 14	5528217
16	12 not 15	2679
17	remove duplicates from 16	2679
18	(2020042* or 2020043* or 202005* or 202006* or 202007* or 202008* or 202009* or 20201* or 2021* or 2022* or 2023*).ed,dt,ez.	5150248
19	17 and 18	947

Table S2 a – Quality rating of the included studies using the consensus-based standards for the selection of health status measurement instruments (COSMIN) subscales for reliability, validity, and responsiveness.

	Reliability		Known-groups validation	Responsiveness	
**Study ID**	**Reliability**	**Measurement error**		**Within-group**	**Between-groups**
Alijnovic et al., 2020	very good	-	very good	-	-
Alis et al., 2018	adequate	-	-	-	-
Aljinovic et al., 2022	-	-	doubtful	-	-
Barun et al., 2021	adequate	-	-	-	-
Bethers et al., 2021	-	-	-	adequate	-
Blain et al., 2019	adequate	-	-	-	-
Buran Cirak et al., 2021	-	-	-	very good	very good
Calvo-Lobo et al., 2017	-	-	-	-	-
Can et al., 2021	-	-	very good	-	-
Chan et al., 2012	inadequate	-	adequate	-	-
Chen et al., 2020	doubtful	-	-	-	-
Creze et al., 2017	-	-	doubtful	-	-
Dieterich et al., 2017	doubtful	adequate	-	-	-
Ding., 2019	-	-	adequate	doubtful	-
Dones et al., 2021	very good	-	very good	-	-
Ertekin et al., 2021	-	-	very good	-	very good
Gao., 2019	adequate	-	very good	-	-
Gao et al., 2020	-	-	very good	doubtful	very good
Griefahn., 2017	-	-		doubtful	-
Griefahn et al., 2021	-	-	-	very good	very good
Güler et al., 2022	-	-	-	doubtful	very good
Gunaydin et al., 2022	-	-	-	adequate	adequate
Heizelmann et al., 2017	-	-	adequate	-	-
Hvedstrup et al., 2020	-	-	very good	-	-
Ishikawa et al., 2017	-	-	very good	-	very good

Ishikawa et al.,2020	-	-	very good	-	-
Karayol et al., 2021	-	-	adequate	-	-
Kelly 2018	very good	-	-	-	-
Kisilewicz et al., 2020	-	-	-	very good	-
Koppenhaver., 2018	very good	very good	-	-	-
Koppenhaver., 2020	-	-	very good	-	-
Koppenhaver et al., 2022	-	-	-	-	very good
Kozinc et al., 2020	very good	-	-	-	-
Kumamoto et al., 2021	adequate	-	-	very good	-
Landen Ludvigsson et al., 2016	very good	very good	very good	-	very good
Langévin et al., 2011	doubtful	-	very good	-	-
Leong et al., 2013	adequate	very good	-	-	-
Leong et al., 2016	-	-	adequate	-	-
Liang et al., 2021	-	-	adequate	-	-
Ma et al., 2020	adequate	-	-	-	-
Masaki et al., 2017	-	-	very good	-	-
Masaki et al., 2019	-	-	adequate	-	-
Moreau et al., 2016	adequate	-	-	-	-
Murillo et al., 2019	very good	-	very good	-	-
Nagai et al., 2020	very good	very good	-	-	-
Peolsson et al., 2008	-	-	very good	very good	-
Peolsson et al., 2010	adequate	-	-	-	-
Peolsson et al., 2013a	-	-	very good	-	-
Peolsson et al., 2015	-	-	very good	-	-
Peterson et al., 2019	doubtful	-	-	-	-
Pimentel-Santos et al., 2021	adequate	-	-	-	-
Pinto et al., 2022	-	-	very good	-	-
Qazi et al., 2022	adequate	adequate	-	-	-
Rahnama et al., 2018	-	-	adequate	-	
Sakaki et al., 2022	doubtful	-	-	doubtful	-
Sanchez-Infante et al., 2021	adequate	-	-	adequate	adequate

Sawada et al., 2020	very good	-	-	-	-
Shimoyama et al., 2021	very good	-	-	-	-
Takla et al., 2016	-	-	very good	-	-
Tamartash et al., 2022	-	-	-	inadequate	-
Tamartash et al., 2023	-	-	inadequate	-	-
Tas et al., 2018	-	-	adequate	-	-
Turo et al., 2015	-	adequate	-	-	-
Valero-Calero et al., 2021	-	-	very good	-	-
Valero-Calero et al., 2022	-	-	-	adequate	adequate
Vatovec et al., 2022	-	-	-	adequate	-
Wachi et al., 2022	-	-	-	adequate	-
Wada et al., 2019	Very good	-	-	-	-
Wang et al., 2020	adequate	very good	-	-	-
Weber et al., 2022	-	-	-	-	very good
Wong et al., 2016	-	-	-	very good	very good
Xie et al., 2019	very good	very good	-	-	-
Xu et al., 2018	-	-	-	very good	adequate
Yamamoto et al., 2017	very good	-	-	-	-
Yamaura et al., 2021	adequate	-	-	very good	-
Yurdakul et al., 2019	-	-	-	very good	-
Zhang et al., 2019	adequate	adequate	-	-	-

Tables S2 b–e – COSMIN Boxes used for reliability, validity, and responsiveness subscales. COSMIN methodology is a modular system that needs to be adapted according to the review purposes [3; 4; 5]. We considered reliability (boxes 6 and 7), construct validation (box 9b) and responsiveness (boxes 10c AND 10d) subscales. Boxes 1 to 5, 8, 9a, 10a and 10b were omitted.

**Table S2 b. Box 6. Reliability**					
**Design requirements**	**Very good**	**Adequate**	**Doubtful**	**Inadequate**	**NA**
1. If patients were implicated in the study, were patients stable in the interim period on the construct to be measured?	Evidence if patients were stable	Assumable that patients were stable	Unclear if patients were stable	Patients were NOT stable	
2. If a treatment was applied, was the time interval measurements appropriate?	Time interval appropriate		Doubtful whether time interval was appropriate or time interval was not stated	Time interval NOT appropriate	
3. Were the test conditions similar for the measurements? e.g. type of administration, environment, instructions.	Test conditions were similar (evidence provided)	Assumable that test conditions were similar	Unclear if test conditions were similar	Test conditions were NOT similar	
**Statistical methods**	**Very good**	**Adequate**	**Doubtful**	**Inadequate**	**NA**
4. Was an intraclass correlation coefficient (ICC) single measure calculated? For intra-rater reliability, was an ICC Model 3 Form 1 calculated?	ICC calculated and model or formula of the ICC is described	ICC calculated but model or form of the ICC not described.	No information of which type of ICC was used and the conditions for measurements are unclear.	No ICC single measure or ICC Model 3 Form 1 calculated	
**Other**	**Very good**	**Adequate**	**Doubtful**	**Inadequate**	**NA**
5. Were there any other important flaws in the design or statistical methods of the study?	No other important methodological flaws		Other minor methodological flaws	Other important methodological flaws	

**Table S2 c. Box 7. Measurement error**					
**Design requirements**	**Very good**	**Adequate**	**Doubtful**	**Inadequate**	**NA**
1. If patients were implicated in the study, were patients stable in the interim period on the construct to be measured?	Evidence if patients were stable	Assumable that patients were stable	Unclear if patients were stable	Patients were NOT stable	
2. If a treatment was applied, was the time interval between the measurements appropriate?	Time interval appropriate		Doubtful whether time interval was appropriate or time interval was not stated	Time interval NOT appropriate	
3. Were the test conditions similar for the measurements? e.g. type of administration, environment, instructions.	Test conditions were similar (evidence provided)	Assumable that test conditions were similar	Unclear if test conditions were similar	Test conditions were NOT similar	
**Statistical methods**	**Very good**	**Adequate**	**Doubtful**	**Inadequate**	**NA**
4. Was the Standard Error of Measurement (SEM), Minimum Detectable Change (MDC), Limits of Agreement (LoA) calculated?	SEM, MDC, or LoA calculated	Possible to calculate SEM, MDC or LoA from the data presented.		SEM, MDC or LoA calculated based on SD from another population	NA
**Other**	**Very good**	**Adequate**	**Doubtful**	**Inadequate**	**NA**
5. Were there any other important flaws in the design or statistical methods of the study?	No other important methodological flaws		Other minor methodological flaws	Other important methodological flaws	

Table S2 d. Box 9. Hypothesis testing for construct validity					
**9b. Comparison between subgroups (discriminative or known-groups validity)**					
**Design requirements**	**Very good**	**Adequate**	**Doubtful**	**Inadequate**	**NA**
1. Was an adequate description provided of important characteristics of the subgroups?	Adequate description of the important characteristics of the subgroups	Adequate description of most of the important characteristics of the subgroups	Poor of no description of the important characteristics of the subgroups		
**Statistical methods**	**Very good**	**Adequate**	**Doubtful**	**Inadequate**	**NA**
2. Were design and statistical methods adequate for the hypotheses to be tested?	Statistical methods applied appropriate	Assumable that statistical methods were appropriate	Statistical methods applied NOT optimal	Statistical methods applied NOT appropriate	
**Other**	**Very good**	**Adequate**	**Doubtful**	**Inadequate**	**NA**
3. Were there any other important flaws in the design or statistical methods of the study?	No other important methodological flaws		Other minor methodological flaws	Other important methodological flaws	

Table S2 e. Box 10. Responsiveness					
**10c. Construct approach: (i.e. hypotheses testing: comparison between subgroups)**					
**Design requirements.**	**Very good**	**Adequate**	**Doubtful**	**Inadequate**	**NA**
1. Was an adequate description provided of important characteristics of the subgroups?	Adequate description of the important characteristics of the subgroups	Adequate description of most of the important characteristics of the subgroups	Poor or no description of the important characteristics of the subgroups		
**Statistical methods**	**Very good**	**Adequate**	**Doubtful**	**Inadequate**	**NA**
2. Were design and statistical methods adequate for the hypotheses to be tested?	Statistical methods applied appropriate	Assumable that statistical methods were appropriate	Statistical methods applied NOT optimal	Statistical methods applied NOT appropriate	
**Other**	**Very good**	**Adequate**	**Doubtful**	**Inadequate**	**NA**
3. Were there any other important flaws in the design or statistical methods of the study?	No other important methodological flaws		Other minor methodological flaws	Other important methodological flaws	
**10d. Construct approach: (i.e. hypotheses testing: before and after intervention)**					
**Design requirements**	**Very good**	**Adequate**	**Doubtful**	**Inadequate**	**NA**
1. Was an adequate description provided of the intervention given?	Adequate description of the intervention	Adequate description of most of the intervention	Poor description of the intervention	NO description of the intervention	
**Statistical methods**	**Very good**	**Adequate**	**Doubtful**	**Inadequate**	**NA**
2. Were design and statistical methods adequate for the hypotheses to be tested?	Statistical methods applied appropriate	Assumable that statistical methods were appropriate	Statistical methods applied NOT optimal	Statistical methods applied NOT appropriate	
**Other**	**Very good**	**Adequate**	**Doubtful**	**Inadequate**	**NA**
3. Were there any other important flaws in the design or statistical methods of the study?	No other important methodological flaws		Other minor methodological flaws	Other important methodological flaws	

References used for the adapted COSMIN methodology.

1 Portney LG, Watkins MP (2009) Foundations of clinical research: applications to practice. Pearson/Prentice Hall Upper Saddle River, NJ
2 Doi SA, Williams GM (2013) Methods of clinical epidemiology. Springer
3 Mokkink LB, de Vet HCW, Prinsen CAC et al (2018) COSMIN Risk of Bias checklist for systematic reviews of Patient-Reported Outcome Measures. Qual Life Res 27:1171-1179
4 Prinsen CAC, Mokkink LB, Bouter LM et al (2018) COSMIN guideline for systematic reviews of patient-reported outcome measures. Qual Life Res 27:1147-1157
5 Terwee CB, Prinsen CAC, Chiarotto A et al (2018) COSMIN methodology for evaluating the content validity of patient-reported outcome measures: a Delphi study. Qual Life Res 27:1159-1170

Table S3 – **Characteristics of studies included in the meta-analysis.**

First author, year	Ultrasound method<br>Excitation method<br>Measured physical quantity (and units)<br>US model, company, country<br>Type of probe, position, and frequency<br>Clinical/research device	Participants' condition<br>N/group<br>Age (mean, SD)<br>Sex (male: female)	Muscle assessed (and spinal level, if specified)	Patient's position (and muscle state)
**Alis et al., 2018**	Shear wave imaging<br>ARFI<br>Shear modulus (kPa)<br>Aplio Platin. 500, Toshiba, CA<br>Convex, parallel, 1-6 MHz<br>Clinical	Patients with unilateral lumbar disk herniation (LDH)<br>33<br>47.30 ± 11.22 years<br>17:16	Lumbar multifidus, slightly below the level of herniation (varies from L3 to L5).	Prone at rest.
**Aljinovic et al., 2020**	Shear wave imaging<br>ARFI<br>Shear modulus (kPa)<br>Aixplorer, SuperSonic, France<br>Linear, parallel, 2-10 MHz<br>Clinical	Patients who were involved in a car accident and suffered a whiplash Injury/asymptomatic control.<br>75 (whiplash)/75 (controls)<br>43.1±13.5 (whiplash)/46.5±16.3 (controls)<br>32:43 (whiplash)/34:41 (controls)	Upper trapezius (mid-way between shoulder and head).	Seated at rest.
**Aljinovic et al., 2022**	Shear wave imaging<br>ARFI<br>unclear<br>Aixplorer, Supersonic, France<br>Linear, parallel, 2–10MHz<br>Clinical	Whiplash associated disorders (WAD)/asymptomatic controls.<br>99(WAD)/75(controls)<br>41.9 ±13/46.5 ± 16.3<br>unclear/45:55	Upper trapezius.	Seated at rest.
**Barun et al., 2021**	Shear wave imaging<br>ARFI<br>Shear modulus (kPa)<br>Aixplorer, Supersonic, France<br>Linear, parallel, 2–10MHz<br>Clinical	Whiplash associated disorders (WAD).<br>22 (WAD)<br>38.6 ±11<br>10:12	Upper trapezius.	Seated at rest.
**Bethers et al., 2021**	Shear wave imaging<br>ARFI<br>Young modulus (kPa)<br>GE LOGIC S8, GE Healthcare<br>Linear, parralel, 2.4-10MHz<br>Clinical	Asymptomatic participants.<br>60<br>27.1 ± 8.8<br>36:24	Upper trapezius.	Prone at rest.

First author, year	Ultrasound method<br>Excitation method<br>Measured physical quantity (and units)<br>US model, company, country<br>Type of probe, position, and frequency<br>Clinical/research device	Participants' condition<br>N/group<br>Age (mean, SD)<br>Sex (male: female)	Muscle assessed (and spinal level, if specified)	Patient's position (and muscle state)
**Blain et al., 2019**	Shear wave imaging<br>ARFI<br>Young modulus (kPa)<br>Aixplorer, SuperSonic, France<br>Convex, parallel, unclear<br>Clinical	Asymptomatic participants.<br>15<br>24 ± 4<br>09:06	Erector spinae (L4), multifidus (L4).	Prone at rest (task 1), Prone + passive arm elevation (task 2), seated at rest (task 3), seated + passive arm elevation (task 4), seated + passive thigh elevation (task 5).
**Buran Cirak et al., 2020**	Shear wave imaging<br>ARFI<br>Shear modulus (kPa)<br>unclear, unclear<br>Linear, parallel, 2–10MHz<br>Clinical	LBP having real sustained natural apophyseal glides treatment (SNAG)/LBP having Sham SNAG.<br>15 (LBP + SNAG)/15 (LBP + Sham SNAG)<br>21 ± 1.7/20.4 ± 0.5<br>05:10/04:11	Erector spinae (L4) and multifidus (L3).	Prone at rest.
**Calvo-Lobo et al., 2017**	Strain imaging<br>Strain ratio (no units)<br>Manual compression<br>LOGIQ P9, GE Healthcare<br>Linear, unclear, 6-15 MHz<br>Clinical	Patients with nonspecific lumbopelvic Pain (LPP).<br>10<br>26.50 ± 7.00<br>09:01	Active and latent myofascial trigger point (A-MTrPs and L-MTrPs) with regards to control points in the lumbar erector spinae of subjects with LPP.	Prone at rest.
**Can et al., 2021**	Shear wave imaging<br>ARFI<br>Young modulus (kPa)<br>MyLab 9, Esaote, unclear<br>Linear, parallel, 4-15 MHz.<br>Clinical	LBP having a lumbar unilateral disc herniation.<br>22 (hernia side)/22 (non-hernia side)<br>44.23 ± 5.49<br>10:12	Multifidus (lumbar hernia level varying from L3 to S1).	Prone at rest.
**Chan et al., 2012**	Strain imaging<br>TUPS<br>Young modulus (kPa)<br>HDI-5000, Philips, USA<br>Cylindrical, unclear, 5 MHz<br>Research	low back pain (LBP)/asymptomatic controls<br>12 (LBP)/12 (controls)<br>36.6 ± 2.9 (LBP)/25.2± 1.1 (No-LBP)<br>24:00:00	Lumbar multifidus (L4).	Prone at rest, upright at rest, 25° forward stooping, 45° forward stooping.

First author, year	Ultrasound method<br>Excitation method<br>Measured physical quantity (and units)<br>US model, company, country<br>Type of probe, position, and frequency<br>Clinical/research device	Participants' condition<br>N/group<br>Age (mean, SD)<br>Sex (male: female)	Muscle assessed (and spinal level, if specified)	Patient's position (and muscle state)
**Chen et al., 2020**	Shear wave imaging<br>ARFI<br>Shear modulus (kPa)<br>Aixplorer, SuperSonic, France<br>Linear, parallel, 2–10MHz<br>Clinical	Asymptomatic male participants.<br>20<br>18.4 ± 0.7<br>20:00	Thoracolumbar fascia (L3-L4).	Seated at rest.
**Creze et al. 2017**	Shear Wave Imaging<br>ARFI<br>Shear modulus (kPa)<br>Aixplorer, SuperSonic, France<br>Supercurved, parallel, 6-10 MHz<br>Clinical	Cadavers/asymptomatic participants<br>23 (16 healthy adults, 7 cadavers)<br>23<br>7:9	Mulitfidus (MF), longissimus (L), iliocostalis (IC) (L3)	Prone at rest.
**Dieterich et al., 2017**	Shear wave imaging<br>ARFI<br>Shear modulus (kPa)<br>Aixplorer, SuperSonic, France<br>Linear, parallel, 2-10 MHz<br>Clinical	Asymptomatic participants<br>11<br>21.7 ± 3.2<br>07:04	Trapezius, splenius capitis, semispinalis capitis, semispinalis cervicis and multifidus (all at C4).	Prone in resting state (task 1), performing a lift of 1/3 of the head's weight (task 2), a lift of 2/3 of the head's weight (task 3), a lift of full head weight (task 4).
**Ding et al., 2019**	Shear wave imaging<br>ARFI<br>Young modulus (kPa)<br>Aixplorer, SuperSonic, France<br>Linear, parallel, 4-15 MHz<br>Clinical	Inpatients having an arthroscopic surgery/inpatient having upper limbs surgery.<br>22 (arthroplasty)/12 (controls)<br>61.91 ± 8.93<br>6:22 (patients) / 3:12 (controls)	Splenius capitis (C4), upper trapezius (mid-point of a line between the C7 and the middle of the acromion).	Seated at rest.
**Dones et al., 2021**	Strain imaging<br>Body movement<br>Displacement (mm)<br>HS1, Konica Minolta, Tokyo, Japan.<br>Linear, parallel, 5-13 MHz<br>Research	Myofascial pain syndrom/asymptomatic controls<br>5 (MFP)/ 5 (controls)<br>37 ± 6<br>02:08	Trapezius deep fascia (C7).	Seated performing cervical flexion, extension, right lateral flexion, left lateral flexion, right rotation, left rotation.

First author, year	Ultrasound method<br>Excitation method<br>Measured physical quantity (and units)<br>US model, company, country<br>Type of probe, position, and frequency<br>Clinical/research device	Participants' condition<br>N/group<br>Age (mean, SD)<br>Sex (male: female)	Muscle assessed (and spinal level, if specified)	Patient's position (and muscle state)
**Ertekin et al., 2021a**	Shear wave imaging<br>ARFI<br>Young modulus (kPa)<br>RS 80, Samsung, Korea.<br>Linear, parallel, 9-12 MHz<br>Clinical	Women with trigger points in the upper part of the trapezius muscle (myofascial pain; MFP) / asymptomatic women.<br>30 (MFP)/ 30 (controls)<br>20.3 ± 1.7/20.9 ± 2.2<br>00:60	Trapezius (1/2 of the length between the spinous process of C7 and acromion).	Seated at rest.
**Gao et al., 2019**	Shear wave imaging<br>ARFI<br>Young modulus (m/s)<br>ACUSON S3000, SIEMENS, CA<br>Linear, parallel, 4-9 MHz<br>Clinical	Patients with low back somatic dysfunction/asymptomatic controls.<br>20 (som. dysfunction)/9 (controls)<br>28, no SD (som. dysfunction) /26, no SD (controls)<br>15:14	Iliocostalis lumborum (ICL) (L1–L5) with somatic dysfunction and ICL without somatic dysfunction (in patients), healthy ICL (in controls).	Prone at rest (task 1), prone performing Superman spine extension (contraction) (task 2). In patients with somatic dysfunction measures were also done immediately before and after osteopathic manipulative treatment.
**Gao et al., 2020**	Shear wave imaging<br>ARFI<br>Shear wave speed (m/s)<br>ACUSON S3000, Siemens Medical Solutions, USA<br>linear, parallel, 4-9 MHz.<br>Clinical	Adults with asymmetric lumbar somatic dysfunction (som. dysfunction).<br>20 (som. dysfunction)<br>28 ± unclear<br>10:10	Iliocostalis lumborum (L1–L5)	Prone at rest.
**Griefahn et al., 2021**	Strain imaging<br>Body movement<br>Strain and displacement (% and mm)<br>MyLabOne, Esaote, Germany.<br>Linear, parallel, 13 MHz.<br>Research	Asymptomatic<br>45<br>26.31 ± 3.92<br>23:22	thoracolumbar fascia (L 2-L3)	Prone lying on a motorized articulated table that passively moved the trunk.

First author, year	Ultrasound method<br>Excitation method<br>Measured physical quantity (and units)<br>US model, company, country<br>Type of probe, position, and frequency<br>Clinical/research device	Participants' condition<br>N/group<br>Age (mean, SD)<br>Sex (male: female)	Muscle assessed (and spinal level, if specified)	Patient's position (and muscle state)
**Griefhan et al., 2017**	Strain imaging<br>Body movement<br>Displacement (mm)<br>MyLab One, Esaote, Germany<br>Linear, parallel, 6-13 MHz<br>Research	Healthy sportive participants.<br>Foam Roll Group (13)/placebo group (12)/ control group (13)<br>23.34 ± 2.58<br>13:25	Thoracolumbar fascia (L 2-L3).	Seated, performing a thoracolumbar flexion of 30° before and 10 minutes after a foam roll (or placebo) treatment. Controls had no treatment.
**Gunaydin et al., 2022**	Shear wave imaging<br>ARFI<br>Shear modulus (kPa)<br>RS80, Samsung, Korea.<br>Linear, parallel, 9 MHz.<br>Clinical	Participants with rounded shoulders performing a home exercice physiotherapy program for 4 weeks with performance taping (PT) treatment, classic taping (CT) treatment and without taping (controls).<br>15(PT)/14(CT)/10(controls)<br>23.3 ± 3.6/22.5 ± 4.1/23.3 ± 5.4<br>unclear	Upper trapezius (unclear).	Seated at rest.
**Heizelmann et al., 2017**	Shear wave imaging<br>ARFI<br>Shear wave speed (m/s)<br>ACUSON S3000, SIEMENS, Germany<br>Linear, parallel, 4-9 MHz<br>Clinical	Participants over 60 years/ under 60 years<br>> 60 years (24)/≤ 60 years (254)<br>35.51 ± 14.93<br>110:168	Erector spinae (level of iliac crest), trapezius (level of the midclavicular line).	Prone at rest.
**Hvedstrup et al., 2020**	Shear wave imaging<br>ARFI<br>Shear wave speed (m/s)<br>Logic E9, GE Healthcare, UK<br>Linear, parallel, 9 MHz<br>Clinical	Migraine patients with ictal neck pain/ migraine patients without ictal neck pain/ asymptomatic controls.<br>52 (migraine with ictal)/ 48 (migraine without ictal)/46 (controls)<br>median (25–75 percentiles): 44 (29–53) (migraine with ictal)/ 42 (32–52) (migraine without ictal)/42 (31–52) (controls)<br>89%/91%/93%	Trapezius (1/3 of the length between the spinous process of C7 and acromion), splenius capitis (C5) and semispinalis capitis (C5).	Seated at rest.

First author, year	Ultrasound method<br>Excitation method<br>Measured physical quantity (and units)<br>US model, company, country<br>Type of probe, position, and frequency<br>Clinical/research device	Participants' condition<br>N/group<br>Age (mean, SD)<br>Sex (male: female)	Muscle assessed (and spinal level, if specified)	Patient's position (and muscle state)
**Ishikawa et al., 2017**	Strain imaging<br>Manual compression<br>Strain ratio (no units)<br>HI VISION Avius, Hitachi, Japan<br>Linear, unclear, 6-14 MHz, acoustic coupler (Hitachi, Japan)<br>Research	Patients with neck and shoulders complaints/ asymptomatic controls.<br>18 (complaints)/ 10 (controls)<br>25.7 ± 2.9/24.5 ± 3.9<br>9:9/8:4	Trapezius (2 cm distal midpoint C7- acromion), levator scapulae (LS) (2 cm distal to scapula sup. angle - midpoint C1 - C4), rhomboid (midpoint scapula inf. angle - T3).	Seated at rest, before and after the typing task.
**Ishikawa et al., 2020**	Strain imaging<br>Manual compression<br>Strain ratio (no units)<br>HI VISION Avius, Hitachi, Japan<br>Linear, unclear, 6-14 MHz, acoustic coupler (Hitachi, Japan)<br>Research	Patients with symptomatic tears/patients with asymptomatic tears/healthy controls.<br>13 (symptomatic)/10 (asymptomatic)/9 (controls)<br>66 ±7/72 ± 8/62 ± 8<br>10:3/3:7/5:7	Trapezius (2 cm distal midpoint C7- acromion), levator scapulae (2 cm distal to scapula sup. angle - midpoint C1 - C4), rhomboid (midpoint scapula inf. angle - T3).	Seated with 0°, 60°, 90° and 120° arm elevation.
**Karayol et al., 2021**	Shear wave imaging<br>ARFI<br>Shear wave speed (m/s)<br>ACUSON S3000, Siemens Medical Solutions, USA.<br>Linear, parallel, 4-9 MHz.<br>Clinical	Participants with fibromyalgia (FM)/asymptomatic controls<br>53 (FM)/ 47 (controls)<br>39 (range, 23–60 years) / 37 (range, 21–58 years).<br>0:53/0:47	Rhomboid major (unclear).	Prone at rest.
**Kelly et al., 2018**	Shear wave imaging<br>ARFI<br>Young modulus (kPa)<br>Aixplorer, SuperSonic, France<br>Linear, parallel, 4-15 MHz<br>Clinical	Asymptomatic participants<br>17 (males)/13 (females)<br>27.87 ± 5.76<br>17:13	Erector spinae (L4).	Prone at rest (task 1), 40% of maximum voluntary contraction (MVC) (task 2) and 80 % MVC (task 3).
**Kisilewicz et al., 2020**	Shear wave imaging<br>ARFI<br>Shear modulus (kPa)<br>LOGIQ S8, GE Healthcare, USA.<br>Linear, parallel, 9 MHz.<br>Clinical	Asymptomatic participants performing eccentric exercises (ECC).<br>14<br>23.2 ± 3.0<br>11:3	Upper trapezius (C7).	Seated at rest.

First author, year	Ultrasound method<br>Excitation method<br>Measured physical quantity (and units)<br>US model, company, country<br>Type of probe, position, and frequency<br>Clinical/research device	Participants' condition<br>N/group<br>Age (mean, SD)<br>Sex (male: female)	Muscle assessed (and spinal level, if specified)	Patient's position (and muscle state)
**Kitamura et al. 2020**	Shear Wave Imaging<br>ARFI<br>Shear modulus (kPa)<br>Aixplorer, SuperSonic, France<br>Superlinear, parralel, 2-10 MHz<br>Clinical	College swimmers with LBP/college swimmers without LBP<br>11 (LBP)/21 (controls)<br>21.1 ± 1.5 (LBP)/20.6 ± 1.5 (controls)<br>32:0	Latissimus dorsi (LD) (10 cm below the acromion).	Seated at rest.
**Koppenhaver et al., 2018**	Shear wave imaging<br>ARFI<br>shear modulus (kPa)<br>Aixplorer, Supersonic, WA<br>Linear, parallel, 2-10 MHz<br>Clinical	Asymptomatic participants<br>36<br>30.4 ± 5.5<br>50%:50%	Erector spinae (ES) (iliocostalis and longissimus muscles) (L4) and multifidus (MF) (L4/L5).	Prone at rest (MF and ES) and at minimal and moderate contraction levels (MF only).
**Koppenhaver et al., 2020**	Shear wave imaging<br>ARFI<br>Shear modulus (kPa)<br>Aixplorer, Supersonic, France<br>Linear, parallel, 2-10 MHz<br>Clinical	LBP/asymptomatic controls<br>60 (LBP)/60 (controls)<br>32.2 ±7.3/31 ± 8<br>40%:60%/57%:43%	Erector spinae (ES) (iliocostalis and longissimus muscles) and multifidus (MF) on the most symptomatic side (right or left) at the most symptomatic level (L3, L4, or L5) for LBP patients and on the right side at the L4 spinal level for controls and patients with bilateral symptoms.	Prone at rest (task 1; ES and MF assessment) and during submaximal contractions (task 2; MF only).
**Koppenhaver et al., 2022**	Shear wave imaging<br>ARFI<br>Shear modulus (kPa)<br>Aixplorer, SuperSonic, France<br>Linear, parallel, 2–10MHz<br>Clinical	Participants with low back pain having dry needling treatment (LBP + DN)/participants with low back pain having SHAM dry needling treatment (LBP + SHAM DN).<br>30 (LBP + DN) / 30 (LBP + SHAM DN)<br>32.2 ± 8.2 / 32.2 ± 6.6<br>16:14/ 20:10	Erector spinae and multifidus (L3-L5)	Prone at rest (ES and multifidus) and during a submaximal contraction (multifidus only).

First author, year	Ultrasound method<br>Excitation method<br>Measured physical quantity (and units)<br>US model, company, country<br>Type of probe, position, and frequency<br>Clinical/research device	Participants' condition<br>N/group<br>Age (mean, SD)<br>Sex (male: female)	Muscle assessed (and spinal level, if specified)	Patient's position (and muscle state)
**Kozinc et al., 2020**	Shear wave imaging<br>ARFI<br>Shear modulus (kPa)<br>Resona 7, Mindray, China<br>linear, parallel, and perpendicular, 3-11 MHz<br>Clinical	Asymptomatic participants.<br>20<br>31.4 ± 9.8<br>8:12	Trapezius (1/2 of the length between the spinous process of C7 and acromion).	Seated at rest (task 1) and performing shoulder abduction at 40° (task 2) and 60° (task 3).
**Kumamoto et al., 2021**	Shear wave imaging<br>ARFI<br>Shear modulus (kPa)<br>Aixplorer, SuperSonic, France<br>Linear, parallel, 2–10MHz<br>Clinical	Fatigue-exercise (F-Ex) group: asymptomatic participants that exercised standing back extension exercises (SBEE) after completing a muscle fatigue-task (Biering Sorensen). Fatigue-non-exercise (F-nEx) group remained standing for the same duration as the F-Ex group without doing SBEE after the muscle fatigue-task. Non-fatigue-exercise (nF-Ex) group exercised five sets without performing the muscle fatigue-task.<br>11 (F-Ex)/11(nF-Ex)/11(F-nEx)<br>26.8 ± 8.6/21.2 ± 3.7/22.5 ± 5.6<br>11:0/11:0/11:0	Multifidus (L5)	Upright at rest.
**Kuo et al. 2013**	Shear Wave Imaging<br>ARFI<br>Shear wave speed (m/s)<br>Acuson S2000, Siemens, Germany<br>linear, unclear, 7–9-MHz<br>Clinical	Chronic neck pain/asymptomatic controls<br>3 (neck pain)/17 (controls)<br>30.8 ± 4.0<br>9:11	Trapezius (TP) (midpoint of occipital protuberance and acromial tip).	Seated at rest.
**Landen Ludvigsson et al., 2016**	Strain imaging<br>Body movement<br>Strain (%)<br>Vivid I, GE Healthcare, Norway<br>Linear, parallel, 12 MHz<br>Research	Whiplash associated disorders (WAD)/asymptomatic controls.<br>36 (WAD)/ 36 (controls)<br>38 ±11 /38 ± 11<br>10:26/10:26	Superior, mid, and deep portions of the upper trapezius (halfway between the acromion and C7).	Upright, performing a scapular elevation task (concentric and eccentric contraction) before and following

First author, year	Ultrasound method<br>Excitation method<br>Measured physical quantity (and units)<br>US model, company, country<br>Type of probe, position, and frequency<br>Clinical/research device	Participants' condition<br>N/group<br>Age (mean, SD)<br>Sex (male: female)	Muscle assessed (and spinal level, if specified)	Patient's position (and muscle state)
				loaded arm abduction.
**Langévin et al., 2011**	Strain imaging<br>Body movement<br>Strain (%)<br>T3000, Terason, USA<br>Linear, parallel, 10 MHz<br>Research	LBP/asymptomatic controls<br>71 (LBP)/50 (controls)<br>44.6 ± 1.8/41.8 ± 2.3<br>38:33/24:26	Thoracolumbar fascia (L 2-L3).	Prone lying on a motorized articulated table that passively moved the trunk.
**Leong et al., 2013**	Shear wave imaging<br>ARFI<br>Shear modulus (kPa)<br>Aixplorer, SuperSonic, France<br>Linear, parallel, 4-15 MHz<br>Clinical	Asymptomatic participants<br>15(males)/13 (females)<br>29.6 ± 13.5<br>15:13	Upper trapezius (mid-way between the angle of acromion and C7).	Seated, in resting position (task 1) and holding a static 30° abduction (task 2).
**Leong et al., 2016**	Shear wave imaging<br>ARFI<br>Shear modulus (kPa)<br>Aixplorer, Supersonic, France<br>Linear, parallel, 4-15 MHz<br>Clinical	Athletes with rotator cuff tendinopathy (RCT)/athletes without RCT.<br>26 with RCT/17 without RCT<br>23.6 ± 3.3/21.7± 3.5<br>43:00:00	Upper trapezius (vertebral level unclear).	Seated during active arm holding at 30° (task 1) and 60° (task 2) of shoulder abduction (active tasks) and with the arm passively positioned at 0° (task 3), 30° (task 4) and 60° (task 5) of shoulder abduction (passive tasks).

First author, year	Ultrasound method<br>Excitation method<br>Measured physical quantity (and units)<br>US model, company, country<br>Type of probe, position, and frequency<br>Clinical/research device	Participants' condition<br>N/group<br>Age (mean, SD)<br>Sex (male: female)	Muscle assessed (and spinal level, if specified)	Patient's position (and muscle state)
**Liang et al., 2021**	Shear wave imaging<br>ARFI<br>Shear modulus (kPa)<br>Aplio 500, Toshiba Medical Systems, Japan.<br>Linear, parallel, 5-14 MHz.<br>Clinical	Participants with back pain presenting myofascial trigger points (MTrPs) on one spinal side and no MTrPs on the other side (controls).<br>30 (MTrPs)<br>47.67 ± 5.79<br>unclear	Trapezius (unclear)	Prone at rest.
**Ma et al., 2020**	Strain imaging and shear wave imaging<br>TUPS and ARFI<br>Young modulus (kPa)<br>Unclear (for TUPS) and Aixplorer (for ARFI), Unclear (for TUPS) and SuperSonic Imaging (for ARFI).<br>128-elements ultrasound transducer with a 20 N in-series load cell (for TUPS); Convex, parallel, 1-6 MHz (for ARFI).<br>Research	Asymptomatic male participants.<br>14<br>23.5 ± 2.9<br>14:0	Unclear (T7 and L1)	Prone at rest.
**Masaki et al., 2017**	Shear wave imaging<br>ARFI<br>Shear modulus (kPa)<br>Aixplorer, SuperSonic, France<br>Linear, parallel, 2-10 MHz<br>Clinical	Medical workers with LBP/asymptomatic medical workers.<br>9 (LBP)/23 (asymptomatic)<br>44.3 ± 13.0/34.7 ± 10.2<br>1:8/8:15	Erector spinae (L3) and multifidus (L4).	Prone at rest.
**Masaki et al., 2019a**	Shear wave imaging<br>ARFI<br>Shear modulus (kPa)<br>Aixplorer, SuperSonic, France<br>Linear, parallel, 2-10 MHz<br>Clinical	Middle-aged and elderly women with LBP/middle-aged and elderly women with LBP history (LBPH)/asymptomatic middle-aged and elderly women.<br>23 (LBP)/ 16 (LBPH)/ 19 (asymptomatic)<br>74.3±6.4/70.3±6.5/72.4±5.4<br>00:23/0:16/0:19	Erector spinae (L3) and multifidus (L4).	Prone at rest.
**Moreau et al., 2016**	Shear wave imaging<br>ARFI<br>Shear modulus (kPa)<br>Aixplorer, SuperSonic, France<br>Linear, parallel, 8 MHz<br>Research	Asymptomatic participants<br>10<br>25.5 ± 2.2<br>06:04	Multifidus (L3-L4) and (L4-L5).	Prone at rest (task 1) and seated on a massage chair with the multifidus in passive stretching (task 2).

First author, year	Ultrasound method<br>Excitation method<br>Measured physical quantity (and units)<br>US model, company, country<br>Type of probe, position, and frequency<br>Clinical/research device	Participants' condition<br>N/group<br>Age (mean, SD)<br>Sex (male: female)	Muscle assessed (and spinal level, if specified)	Patient's position (and muscle state)
**Murillo et al., 2019**	Shear wave imaging<br>ARFI<br>Shear modulus (kPa)<br>LOGIQ S8, GE Healthcare, USA<br>Linear, parallel, 9 MHz<br>Clinical	LBP/asymptomatic controls<br>15 (LBP)/15 (asymptomatic)<br>29.4 ± 10.80/26.71 ± 5.40<br>46.7 % males/53.30 % males	Superficial and deep multifidus (L3-L4).	Prone at rest (superficial and deep MF assessment) (task 1) and prone with 15° steady trunk extension (superficial MF only) (task 2).
**Nagai et al., 2020**	Shear wave imaging<br>ARFI<br>Shear modulus (kPa)<br>Aixplorer, Supersonic, WA<br>Linear, parallel, 5-18 MHz<br>Clinical	Physical active young adult males and females<br>11 males/13 females<br>25.6 ± 4.9/ 23.7 ± 1.9<br>11:13	Upper trapezius, at the distance between C7 and the most lateral part of the acromion process (1) and at 2/3 of the distance from the acromion process (2).	Prone at rest.
**Peolsson et al. 2008**	Strain Imaging<br>Body movement<br>Strain (%) and strain rate (%/s)<br>Vivid 7 Dimension<br>GE Healthcare, Norway.<br>linear, parralel, 12 MHz.<br>Research	female patients with trapezius myalgia and asymptomatic controls<br>14 (TM)/13 (asymptomatic)<br>38 (range:24-48)/43 (range:36-55)<br>0:27	Trapezius (mid-distance on a line from C7 to the edge of acromion).	Seated, performing a 3-cm concentric shoulder elevation before and after pain provocation/ exercise.
**Peolsson et al., 2010**	Strain imaging<br>Body movement (no units)<br>Strain (%) and strain rate (%/s)<br>Vivid 7 Dimension, GE Healthcare, Norway<br>Linear, parallel, 14 MHz<br>Research	University student volunteers.<br>15<br>25 ± 3.8<br>06:09	Trapezius, splenius, semispinalis capitis, semispinalis cervicis, and multifidus (all at C4).	Seated, performing a sub-maximal isometric head extension against manual resistance ("initial contraction phase"), maintaining the position ("plateau phase") and letting go of the external handheld resistance

First author, year	Ultrasound method<br>Excitation method<br>Measured physical quantity (and units)<br>US model, company, country<br>Type of probe, position, and frequency<br>Clinical/research device	Participants' condition<br>N/group<br>Age (mean, SD)<br>Sex (male: female)	Muscle assessed (and spinal level, if specified)	Patient's position (and muscle state)
				("end phase").
**Peolsson et al., 2013a**	Strain imaging<br>Body movement<br>Strain (%) and strain rate (%/s)<br>Vivid 9 Dimension, GE Healthcare, Norway<br>Linear, parallel, 14 MHz<br>Research	Individuals with residual disability 10 years after anterior cervical decompression and fusion (ACDF) for cervical disc disease/10 asymptomatic controls<br>10 (ACDF) / 10 (asymptomatic)<br>60±7.1/60 ±6.5<br>7:3/7:3	Trapezius, splenius, semispinalis capitis, semispinalis cervicis, and multifidus (all at C4).	Upright, performing a single loaded arm lift to 120° while holding a standardized weight in the hand (1 kg females, 2 kg males) (task 1) and 10 repeated loaded arm lift to 90° while holding a standardized weight in the hand (0.5 kg females, 1 kg males). (task 2).
**Peolsson et al., 2015**	Strain imaging<br>Body movement<br>Strain (%) and strain rate (%/s)<br>Vivid 9 Dimension, GE Healthcare, Norway<br>Linear, parallel, 14 MHz<br>Research	Individuals with residual disability 10 years after ACDF for cervical disc disease/10 asymptomatic controls<br>10 (ACDF) / 10 (asymptomatic)<br>60±7.1/60 ±6.5<br>7:3/7:3	Trapezius, splenius, semispinalis capitis, semispinalis cervicis, and multifidus (all at C4).	Seated, performing a 20° head extension against resistance (2 kg males, 1 kg females).
**Peterson et al., 2019**	Strain imaging<br>Body movement<br>Strain (%)<br>Vivid Dimension, GE Healthcare, Norway<br>Linear, parallel, 12 MHz<br>Research	Asymptomatic participants<br>20<br>24 ± 5.3<br>12:08	Trapezius, splenius, semispinalis capitis, semispinalis cervicis, and multifidus (all at C4).	Upright, performing a head extension against a resistance of 10%, 20%, 40%, 60%, and 80% of MVC.

First author, year	Ultrasound method<br>Excitation method<br>Measured physical quantity (and units)<br>US model, company, country<br>Type of probe, position, and frequency<br>Clinical/research device	Participants' condition<br>N/group<br>Age (mean, SD)<br>Sex (male: female)	Muscle assessed (and spinal level, if specified)	Patient's position (and muscle state)
**Pimentel Santos et al., 2021**	Shear wave imaging<br>ARFI<br>Shear modulus (kPa)<br>Aixplorer v 10, SuperSonic, France.<br>unclear, unclear, unclear<br>Clinical	Asymptomatic participants.<br>17<br>26.5 (23.5 - 32.5) (mean (range))<br>8:9	Multifidus (L3-L4)	Prone at rest.
**Pinto et al., 2022**	Shear wave imaging<br>ARFI<br>Shear modulus (kPa)<br>Aixplorer, SuperSonic, France.<br>Convex, parallel, 1-6 MHz<br>Clinical	Participants with low back pain /asymptomatic controls<br>78 (LBP) / 73 (controls)<br>46.0 (35.8 - 54) / 48 (30 - 54.5)<br>16:14/ 20:10	Mulitfidus (L4-L5 and L5-S1)	Prone at rest.
**Qazi et al., 2022**	Shear wave imaging<br>ARFI<br>Strain ratio (no units)<br>Aixplorer, SuperSonic, France., SuperSonic, France.<br>Linear, parallel, 5-14 MHz<br>Clinical	Participants with unilateral lumbar radicular pain/asymptomatic controls.<br>15 (radicular pain)/10 (controls)<br>38.53 ± 8.13<br>unclear/unclear	Multifidus (unclear)	Prone at rest.
**Rhanama et al., 2018**	Strain imaging<br>Body movement<br>Strain (%) and strain rate (%/s)<br>Vivid-I, GE Healthcare, Norway<br>Linear, parallel, 12 MHz<br>Research	36 (including two drop-outs) (WAD)/36 (asymptomatic)<br>37 ± 10.78/37 ± 10.54<br>26:10/26:10<br>26:10/26:10	Trapezius, splenius, semispinalis capitis, semispinalis cervicis, and multifidus (all at C4).	Prone on a table performing a head extension with a load (4 kg males, 2 kg females) applied around the head.
**Sakaki et al., 2022**	Shear wave imaging<br>ARFI<br>Shear modulus (kPa)<br>unclear, Unclear<br>Unclear, parallel, unclear<br>Clinical	Participants that underwent arthroscopic rotator cuff repair (ARCR).<br>8<br>61.5 ± 9.4<br>8:0	Trapezius (unclear).	Seated performing a 30° maximum voluntary isometric shoulder abduction in the scapular plane.

First author, year	Ultrasound method<br>Excitation method<br>Measured physical quantity (and units)<br>US model, company, country<br>Type of probe, position, and frequency<br>Clinical/research device	Participants' condition<br>N/group<br>Age (mean, SD)<br>Sex (male: female)	Muscle assessed (and spinal level, if specified)	Patient's position (and muscle state)
**Sanchez-Infant et al., 2021**	Strain imaging and shear wave imaging<br>Manual compression and ARFI<br>Strain ratio (no units) and shear modulus (kPa)<br>LOGIC S8 (for TUPS) and unclear (for ARFI), GE, USA (for TUPS) and SIEMENS, unclear (for ARFI)<br>linear, parallel, 4-15 MHz (for TUPS) and linear, parallel, 4-9 MHz (for ARFI).<br>Clinical	Healthy young people with latent trigger points on their upper trapezius (dominant side) having one session of dry needling (DN) or one session of SHAM DN treatment.<br>19 (DN)/ 24 (Sham DN)<br>23.68 ± 3.41/ 24.45 ± 3.48<br>19:8/18:6	Upper trapezius (middle third).	Seated at rest.
**Sawada et al., 2020**	Strain imaging<br>Manual compression<br>Strain ratio (no units)<br>HIVISION Preirus, Hitachi, Japan.<br>linear, parallel, 5-18 MHz, acoustic coupler (Hitachi, Tokyo, Japan).<br>Clinical	Asymptomatic male participants.<br>20<br>20.3 ± 0.6<br>20:0	Trapezius (1/2 of the length between the spinous process of C7 and acromion).	Seated at rest.
**Shimoyama et al., 2021**	Strain imaging<br>Manual compression<br>Strain ratio (no units)<br>EUB-7500, Hitachi, Japan.<br>linear, parallel, 6-14 MHz, acoustic coupler (Hitachi, Tokyo, Japan).<br>Clinical	Asymptomatic male participants.<br>22<br>27.6 ± 6.69<br>22:0	Trapezius (1/3 of the distance between the spine and the acromion, midway between C7 and the superior medial scapular angle).	Seated at rest.
**Takla et al., 2016**	Vibration elastography<br>Controlled external vibration<br>Strain ratio (no units)<br>EUB-7500, Hitachi, Japan<br>Linear, unclear, unclear<br>Research	Participants with active myofascial trigger points (A-MTrPs) or passive myofascial trigger points (P-MTrPs).<br>50 participants (compiling 153 A-MTrPs and 159 P-MTrPs).<br>34.28 ± 6.17/ 35.8 ± 5.91<br>unclear	A-MTPs and P-MTPs in the longissimus thoracis (T10-T11 and L1) with regards to normal surrounding tissue of the longissimus thoracis.	Rest lying on its side.
**Tamartash et al., 2022**	Strain imaging<br>Manual compression<br>Elastic modulus coefficient (Pa)<br>Sonix TOUCH, Ultrasonic, Canada.<br>linear, parallel, 5 - 14 MHz<br>Research	Participants with low back pain having myofascial release treatment (LBP + MFR)/participants with low back pain having electrotherapy treatment (LBP + ET).<br>16 (LBP + MFR) / 16 (LBP + ET)<br>40.31 ± 5.45/ 42.19 ± 5.03<br>unclear/unclear	Thoracolumbar fascia (L 2-L3 and L4-L5)	Prone at rest.

First author, year	Ultrasound method<br>Excitation method<br>Measured physical quantity (and units)<br>US model, company, country<br>Type of probe, position, and frequency<br>Clinical/research device	Participants' condition<br>N/group<br>Age (mean, SD)<br>Sex (male: female)	Muscle assessed (and spinal level, if specified)	Patient's position (and muscle state)
**Tamartash et al., 2023**	Strain imaging<br>Manual compression<br>Elastic modulus coefficient (kPa)<br>SONON, Guro-dong, South Korea<br>linear, parallel, 5 - 14 MHz.<br>Research	Participants with low back (LBP)/asymptomatic controls (controls).<br>68 (LBP) / 63 (controls)<br>40.2 ± 5.3/ 41.7 ± 4.9<br>33:35/32:31	Thoracolumbar fascia (L 2-L3 and L4-L5)	Prone at rest.
**Tas et al., 2018**	Shear wave imaging<br>ARFI<br>Shear wave speed (m/s)<br>ACUSON S3000, SIEMENS, CA<br>Linear, parallel, 4-9 MHz<br>Clinical	Patients with chronic neck pain/ asymptomatic participants.<br>35 (neck pain)/35 (asymptomatic)<br>35.7 ± 8.3/35.2 ± 9.0<br>unclear	Upper trapezius (2 cm lateral to C7) and splenius capitis (2 cm lateral to C4), levator spinae.	Prone at rest.
**Turo et al., 2015**	Vibration sonoelastography<br>Controlled external vibration<br>Mechanical heterogeneity index (no units)<br>Sonix RP, Ultrasonix Corp, CA<br>Unclear, unclear, 10 MHz<br>Research	Patients with chronic myofascial pain and at least one palpable active myofascial trigger point (A-MTrP).<br>48<br>35 ± 13<br>18:30	In the upper trapezius approximately midway between the cervical vertebrae and the acromion process: (1) A-MTPs that changed from active to latent or palpably normal tissue after treatment (defined as "responded"). (2) A-MTPs that changed from active to palpably normal tissue after treatment (defined as "resolved"). (3) A-MTPs that responded but did not resolve ("active to latent").	unclear
**Valera-Calero et al., 2021**	Shear wave imaging<br>ARFI<br>Shear wave speed (m/s)<br>APLIO A, CANON<br>linear, parallel, 5 - 14 MHz.<br>Clinical	Participants with chronic neck pain/ asymptomatic participants.<br>19 (neck pain)/ 34 (asymptomatic)<br>21.5 ± 3.2/ 21.9 ± 5.9<br>unclear/unclear	Upper trapezius (unclear).	Prone at rest.

First author, year	Ultrasound method<br>Excitation method<br>Measured physical quantity (and units)<br>US model, company, country<br>Type of probe, position, and frequency<br>Clinical/research device	Participants' condition<br>N/group<br>Age (mean, SD)<br>Sex (male: female)	Muscle assessed (and spinal level, if specified)	Patient's position (and muscle state)
**Valera-Calero et al., 2022**	Shear wave imaging<br>ARFI<br>Shear wave speed (m/s)<br>APLIO A, CANON<br>linear, parallel, 5 - 14 MHz.<br>Clinical	Participants with chronic neck pain having dry needling (DN) / asymptomatic participants having SHAM DN<br>32 (DN)/ 28 (SHAM DN)<br>22.3 ± 7.9/ 21.4 ± 2.3<br>unclear/unclear	Upper trapezius (unclear).	Prone at rest.
**Vatovec et al., 2022**	Shear wave imaging<br>ARFI<br>Shear modulus (kPa)<br>Resona 7, Mindray, China.<br>Unclear, parallel, unclear.<br>Clinical	Asymptomatic participants.<br>22<br>22.5 ± 3.0<br>11:11	Superficial and deep multifidus, deep erector spinae (L4).	Prone at rest (superficial MF and ES, deep MF) (task 1), prone at rest after isometric trunk contraction (fatigue exercise) (task 2), prone during low level (task 3) and high level of contractions (task 4) (superficial MF and ES only).
**Vining et al., 2022**	Strain Imaging<br>t3000<br>Terason, Burlington, USA.<br>Linear, parallel, 10 MHz<br>Research	LBP having chiropractic care for 8 weeks.<br>20<br>41.1 ± 12.6<br>9:11	Thoracolumbar fascia (L2-L3)	Prone-lying on a mototorized articluated table that passively moved the trunk.
**Wachi et al., 2022**	Strain imaging<br>manual compression<br>Strain ratio (no units)<br>SSD-3500SV, FujiFilm, Tokyo.<br>Linear, parallel, 7.5 MHz, acoustic coupler (FujiFilm, Tokyo).<br>Clinical	Participants with low back pain (LBP) having capacitive and resistive electric transfer therapy (CRet)/ LBP participants having SHAM CRet.<br>12 (CRet) / 12 (SHAM CRet)<br>34.3 ± 8.7 / 32.5 ± 7.5<br>12:0/12:0	Multifidus (unclear).	unclear

First author, year	Ultrasound method<br>Excitation method<br>Measured physical quantity (and units)<br>US model, company, country<br>Type of probe, position, and frequency<br>Clinical/research device	Participants' condition<br>N/group<br>Age (mean, SD)<br>Sex (male: female)	Muscle assessed (and spinal level, if specified)	Patient's position (and muscle state)
**Wada et al. 2019**	Shear Wave Imaging<br>ARFI<br>Unclear (kPa)<br>Aixplorer, SuperSonic, France<br>Linear, parallel, 2-10 MHz<br>Clinical	Patients with frozen shoulder being in the freezing (phase 1) or frozen phase (phase 2).<br>15 (freezing) /17 (frozen)<br>54.9 ± 9.8/63.4 ± 8.1<br>5:9/8:9	Middle trapezius (vertebrae level unclear).	Seated at rest.
**Wang et al., 2020**	Shear wave imaging<br>ARFI<br>Shear wave speed (m/s)<br>Aixplorer, SuperSonic, France.<br>Linear, parallel, 4-15 MHz.<br>Clinical	Asymptomatic participants.<br>20<br>unclear<br>unclear	Trapezius (1/2 between the spinous process of C7 and acromion).	Seated, in resting position (task 1) and in 90° passive abduction (task 2).
**Weber et al., 2022**	Strain imaging<br>Body movement<br>Displacement and relative displacement (mm)<br>SonidoSmart Plus, Zimmer Medizin System, Germany.<br>Linear, parallel, 16 MHz.<br>Clinical	Female soccer athletes.<br>23 (IAMT) / 22 (CG) / 22 (PG)<br>21.2 ± 4.1 / 20.9 ± 3.6 / 20.8 ± 4.1<br>0:23/ 0:22/0:22	Erector spinae (ES), superficial fascia (SF), superior (SL) and deep (DL) lamina of the thoracolumbar fascia (TLF), (all at L 2-L3).	Prone lying on a motorized articulated table that passively moved the trunk.
**Wong et al., 2016**	Strain imaging<br>Body movement<br>Stiffness index (mm, N/mm and no units)<br>T3000, Terason, USA<br>Linear, parallel, 5-12 MHz<br>Research	Asymptomatic male participants.<br>10<br>22.8 ± 2.0<br>10:00	Junction of the latissimus dorsi muscle and the thoracolumbar fascia.	Prone, performing a "press-down" task from resting (0%) to MVC (100%) followed by a ramped relaxation before and after myofascial release treatment.
**Xie et al., 2019**	Shear wave imaging<br>ARFI<br>Shear modulus (kPa)<br>Aixplorer, SuperSonic, France<br>Linear, parallel, 2-10 MHz<br>Research	Asymptomatic participants.<br>16<br>29.3 ± 9.8<br>08:08	Upper trapezius, anterior upper trapezius, posterior upper trapezius, spinalis capitis, semispinalis capitis and cervicis, multifidus, middle and lower	Seated at rest.

First author, year	Ultrasound method<br>Excitation method<br>Measured physical quantity (and units)<br>US model, company, country<br>Type of probe, position, and frequency<br>Clinical/research device	Participants' condition<br>N/group<br>Age (mean, SD)<br>Sex (male: female)	Muscle assessed (and spinal level, if specified)	Patient's position (and muscle state)
			trapezius, levator scapular.	
**Xu et al., 2018**	Shear wave imaging<br>ARFI<br>Young modulus (kPa)<br>Aixplorer, SuperSonic, France<br>Linear, parallel, 2-10 MHz<br>Clinical	LBP with primary osteoporosis (POP) having calcium treatment + moxibustion treatment for 3 days a week for 4 weeks/LBP with POP having only calcium treatment for 4 weeks.<br>32 (including 3 dropouts) (moxhi + Ca)/31 (including 4 dropouts) (Ca only)<br>65.16 ± 6.82/63.90 ± 7.59<br>3:29/2:29	Right and left multifidus (L4).	Prone at rest.
**Yamamoto et al., 2017**	Vibration sonoelastography<br>Controlled external vibration<br>Shear wave speed (m/s)<br>EUB-8500, Hitachi<br>Linear, parallel, 6.5 MHz<br>Research	Asymptomatic male participants.<br>23<br>28.8 (range: 21- 42)<br>23:00	Trapezius (1/3 of the length between the spinous process of C7 and acromion).	Seated at rest.
**Yamaura et al., 2021**	Shear wave imaging<br>ARFI<br>Shear modulus (kPa)<br>Aplio 500, Toshiba Medical Systems, Japan.<br>Linear, parallel, 4-13 MHz<br>Clinical	Baseball players' throwing shoulder at 3 time points: before, immediately after, and 24 hours after a throwing session of 100 pitches.<br>14<br>26.6 (24-33) (mean (range))<br>unclear	Middle trapezius and rhomboideus (midway of T2-T4 and scapula), lower trapezius (5 cm below the root of the scapular spine).	Seated at rest.
**Yurdakul et al. 2019**	Strain Imaging<br>Manual compression<br>Strain ratio (no units)<br>Aplio 500, Toshiba, Japan.<br>Unclear<br>Clinical	Patients with low back pain treated for a period of 3 weeks with hot pack and TENS (H+T, group 1), HP, TENS and therapeutic ultrasounds (H+T+U, group 2), and asymptomatic controls (group 3).<br>23 (H+T)/23 (H+T+U)/23 (controls)<br>51.7±10.7/48.6±11.0/46.3 ±10.7<br>11:12/11:12/11:12	Multifidus (L4).	Prone at rest.

First author, year	Ultrasound method<br>Excitation method<br>Measured physical quantity (and units)<br>US model, company, country<br>Type of probe, position, and frequency<br>Clinical/research device	Participants' condition<br>N/group<br>Age (mean, SD)<br>Sex (male: female)	Muscle assessed (and spinal level, if specified)	Patient's position (and muscle state)
**Zhang et al., 2019**	Shear wave imaging<br>ARFI<br>Shear modulus (kPa)<br>Aixplorer, SuperSonic, France<br>Linear, parallel, 2-10 MHz<br>Clinical	Asymptomatic male participants.<br>20<br>23.1 ± 2.7<br>20:00	Upper trapezius (mid-way between the angle of acromion and C7).	Seated performing 0° (task 1) and 50° (task 2) cervical flexion.

Table S4 a – Pooled intraclass correlation coefficient (ICC) by study included in the meta-analysis.

TABLE S4 a. RELIABILITY			
Test-retest for strain imaging			
ID	**Pooled ICC**	**Pooled 95 Upper CI**	**Pooled 95 Lower CI**
**Landen Ludvigsson, 2016**	0.81	0.24	0.96
**Peollson, 2010**	0.96	0.87	0.99
Test-retest for shear wave imaging			
ID	**Pooled ICC**	**Pooled 95 Upper CI**	**Pooled 95 Lower CI**
**Dieterich, 2017**	0.98	0.94	0.99
**Koppenhaver, 2018**	0.58	0.47	0.67
**Kozinc et al., 2020**	0.69	0.52	0.81
**Leong, 2013**	0.81	0.53	0.93
**Qazi et al., 2022**	0.92	0.8	0.97
**Sanchez-Infante et al., 2021**	0.76	-0.74	0.99
**Xie, 2019**	0.78	0.70	0.84
**Zhang, 2019**	0.86	0.78	0.91
Test-retest for vibration sonoelastography			
ID	**Pooled ICC**	**Pooled 95 Upper CI**	**Pooled 95 Lower CI**
**Turo, 2015**	0.98	0.96	0.99
Intra-rater reliability for strain imaging			
ID	**Pooled ICC**	**Pooled 95 Upper CI**	**Pooled 95 Lower CI**
**Chan, 2012**	0.88	-	-
**Dones, 2021**	0.86	0.71	0.94
**Langévin, 2011**	0.98	-	-
**Ma et al., 2020**	0.87	0.81	0.92
**Peterson, 2019**	0.64	0.47	0.76
**Sawada et al., 2020**	0.94	0.80	0.98
**Shimoyama et al., 2021**	0.60	-0.71	0.98
Intra-rater reliability for shear wave imaging			
ID	**Pooled ICC**	**Pooled 95 Upper CI**	**Pooled 95 Lower CI**
**Alijnovic, 2020**	0.96	0.90	0.98
**Barun, 2021**	0.86	0.78	0.91
**Blain, 2019**	0.71	0.65	0.77
**Chen, 2020**	0.91	-0.84	1.00
**Gao, 2019**	0.97	0.91	0.99
**Kelly, 2018**	0.61	0.32	0.80

**Kitamura et al., 2020**	0.76	-	-
**Koppenhaver, 2018**	0.71	0.55	0.82
**Kozinc et al., 2020**	0.86	0.67	0.95
**Leong, 2013**	0.95	-0.71	1.00
**Moreau, 2016**	0.91	0.69	0.97
**Murillo, 2019**	0.88	0.66	0.96
**Nagai, 2020**	0.73	0.36	0.89
**Pimentel-Santos et al. 2021**	0.90	-0.92	1.00
**Qazi et al., 2022**	0.95	0.49	1.00
**Sanchez-Infante et al., 2021**	0.80	-0.87	1.00
**Sasaki et al., 2022**	0.99	0.99	1.00
**Wang et al., 2020**	0.95	0.81	0.99
**Xie, 2019**	0.77	0.72	0.82
**Yamaura et al., 2021**	0.84	0.80	0.87
Intra-rater reliability for vibration sonoelastography			
**Yamamoto, 2017**	0.91	0.76	0.96

Inter-rater reliability for Strain imaging

ID	**Pooled ICC**	**Pooled 95 Upper CI**	**Pooled 95 Lower CI**
**Ma et al., 2020**	1.00	0.99	1.00
**Shimoyama et al., 2021**	0.62	0.27	0.83
Inter-rater reliability for shear wave imaging			
ID	**Pooled ICC**	**Pooled 95 Upper CI**	**Pooled 95 Lower CI**
**Alis, 2018**	0.94	0.9	0.96
**Barun, 2021**	0.81	0.72	0.88
**Gao, 2019**	0.8	0.62	0.89
**Kozinc et al., 2020**	0.74	0.45	0.89
**Kumamoto et al., 2021**	0.93	0.82	0.97
**Leong, 2013**	0.81	0.69	0.88
**Moreau, 2016**	0.91	0.69	0.97
**Wang et al., 2020**	0.94	0.85	0.98
**Yamaura et al., 2021**	0.77	0.68	0.83
**Zhang, 2019**	0.97	-0.91	1.00
Inter-rater reliability for vibration sonoelastography			
ID	**Pooled ICC**	**Pooled 95 Upper CI**	**Pooled 95 Lower CI**
**Yamamoto, 2017**	0.83	0.56	0.94

ID: **study identification;** ICC: **intraclass correlation coefficient;** CI: **confidence interval.**

Table S4 b – Pooled standardized mean differences (pSMD) by known-group validation study included in the meta-analysis.

TABLE S4 b. KNOWN – GROUP VALIDATION						
Strain imaging						
ID	**pSMD**	**SE[a]**	**t**	**Sig. (2-tailed)**	**Pooled CI Lower**	**Pooled CI Upper**
**Calvo-Lobo et al., 2017[b]**	1.863	0.5355	3.478	0.001	0.813	2.912
**Chan et al., 2012**	1.620	0.3464	4.676	0.000	0.941	2.298
**Dones et al., 2021**	-0.470	0.0544	-8.638	0.000	-0.576	-0.363
**Ishikawa et al., 2017**	1.017	0.3951	2.573	0.010	0.242	1.791
**Ishikawa et al., 2020**	1.689	0.3098	5.453	0.000	1.082	2.297
**Landen Ludvigsson et al., 2016**	0.053	0.0712	0.739	0.460	-0.087	0.192
**Langévin et al., 2011[b]**	0.535	0.1878	2.851	0.004	0.167	0.903
**Peolsson et al. 2008**	0.286	0.1619	1.766	0.077	-0.031	0.603
**Peolsson et al., 2013a**	-0.186	0.0768	-2.426	0.015	-0.337	-0.036
**Peolsson et al., 2015**	0.217	0.1294	1.677	0.094	-0.037	0.471
**Rhanama et al., 2018**	0.343	0.0619	5.539	0.000	0.221	0.464
**Tamartash et al., 2023**	0.931	0.0382	24.337	0.000	0.856	1.006
Shear wave imaging						
ID	**pSMD**	**SE[a]**	**t**	**Sig. (2-tailed)**	**Pooled CI Lower**	**Pooled CI Upper**
**Alijnovic et al., 2020**	0.903	0.1777	5.081	0.000	0.554	1.251
**Alijnovic et al., 2022 [b]**	1.589	0.1873	8.483	0.000	1.222	1.956
**Can et al., 2021**	0.590	0.2611	2.260	0.024	0.078	1.102
**Creze et al. 2017**	0.480	0.4654	1.032	0.302	-0.432	1.393
**Ding et al. 2019**	2.751	0.7292	3.772	0.000	1.322	4.180
**Ertekin et al., 2021**	1.370	0.0521	26.279	0.000	1.268	1.472
**Gao et al., 2019 [b]**	0.722	0.4124	1.750	0.080	-0.087	1.530
**Gao, 2020 [b]**	0.708	0.3260	2.172	0.030	0.069	1.347
**Heizelmann et al., 2017**	0.346	0.0908	3.811	0.000	0.168	0.524
**Hvedstrup et al., 2020**	0.191	0.1521	1.257	0.209	-0.107	0.489
**Karayol et al., 2021[b]**	0.437	0.2039	2.146	0.032	0.038	0.837
**Koppenhaver et al., 2020**	0.313	0.2591	1.210	0.226	-0.194	0.821
**Kuo et al., 2013 [b]**	1.209	0.6547	1.846	0.065	-0.075	2.492
**Leong et al., 2016**	0.926	0.3054	3.033	0.002	0.328	1.525

**Liang et al., 2021.**	0.379	0.1650	2.298	0.022	0.056	0.703
**Masaki et al., 2017**	0.532	0.3585	1.484	0.138	-0.171	1.234
**Masaki et al., 2019a**	0.412	0.2933	1.406	0.160	-0.163	0.987
**Murillo et al., 2019**	0.327	0.6995	0.467	0.640	-1.044	1.698
**Pinto et al. 2022**	0.090	0.0111	8.083	0.000	0.068	0.112
**Tas et al., 2018**	0.432	0.2547	1.695	0.090	-0.068	0.931
**Valero-Calero et al., 2021**	0.157	0.1428	1.099	0.272	-0.123	0.437
**Wada et al. 2019**	0.063	0.1741	0.364	0.716	-0.278	0.405
Vibration sonoelastography						
ID	**pSMD**	**SE[a]**	**t**	**Sig. (2-tailed)**	**Pooled CI Lower**	**Pooled CI Upper**
**Takla et al., 2016**	1.957	1.0192	1.920	0.055	-0.041	3.954

a. **Knapp-Hartung method is used for standard error adjustment.** b. **Some statistics cannot be computed because this subgroup contains a single record.** pSMD: **pooled standardized mean difference;** ID: **study identification;** SE: **standard error of the pooled effect size.** CI: **confidence interval;** t: **t statistic of the pooled effect size;** Sig.: **P value associated to the t statistic.**

Table S4 c – Pooled standardized mean difference (pSMD) by responsiveness study included in the meta-analysis.

TABLE S4 c. RESPONSIVENESS						
WITHIN RESPONSIVENESS FOR STRAIN IMAGING						
ID	**pSMD**	**SE[a]**	**t**	**Sig. (2-tailed)**	**Pooled CI Lower**	**Pooled CI Upper**
**Griefhan et al. 2017 [b]**	1.107	0.3772	2.935	0.003	0.368	1.846
**Griefhan et al. 2021**	0.754	0.2927	2.575	0.010	0.180	1.327
**Peolsson et al. 2008**	0.680	0.4385	1.550	0.121	-0.180	1.539
**Sanchez-Infante et al., 2021**	1.045	0.0606	17.228	0.000	0.926	1.163
**Tamartash et al. 2022**	0.973	0.0976	9.963	0.000	0.781	1.164
**Vining et al., 2022**	0.091	0.2568	0.353	0.724	-0.413	0.594
**Wachi et al., 2022**	1.212	0.3809	3.182	0.001	0.465	1.958
**Wong et al. 2016**	0.192	0.2775	0.693	0.489	-0.352	0.736
**Yurdakul et al., 2019**	-0.103	0.1032	-0.994	0.320	-0.305	0.100
WITHIN RESPONSIVENESS FOR SHEAR WAVE SPEED IMAGING						
ID	**pSMD**	**SE[a]**	**t**	**Sig. (2-tailed)**	**Pooled CI Lower**	**Pooled CI Upper**
**Bethers et al. 2021**	0.466	0.0912	5.110	0.000	0.287	0.644
**Buran Cirak et al. 2021**	1.719	0.0568	30.278	0.000	1.608	1.830
**Ding et al. 2019**	2.082	0.5320	3.913	0.000	1.039	3.124
**Gao, 2020**	0.862	0.3306	2.606	0.009	0.214	1.509
**Gunaydin et al., 2022**	0.370	0.0665	5.557	0.000	0.239	0.500
**Kisilewicz et al., 2020 [b]**	4.525	0.7131	6.346	0.000	3.128	5.923
**Kumamoto et al., 2021**	1.365	0.1051	12.989	0.000	1.159	1.571
**Sasaki et al., 2022**	0.861	0.2845	3.028	0.002	0.304	1.419
**Valero-Calero et al., 2022**	-0.017	0.0685	-0.254	0.800	-0.152	0.117
**Vatovec et al., 2022**	0.221	0.0683	3.240	0.001	0.087	0.355
**Xu et al. 2018**	3.003	0.5543	5.418	0.000	1.917	4.089
**Yamaura et al., 2021**	0.808	0.0651	12.407	0.000	0.681	0.936
BETWEEN RESPONSIVENESS FOR STRAIN IMAGING						
ID	**pSMD**	**SE[a]**	**t**	**Sig. (2-tailed)**	**Pooled CI Lower**	**Pooled CI Upper**
**Griefhan et al. 2021**	0.394	0.1832	2.153	0.031	0.035	0.753

**Sanchez-Infante et al., 2021**	0.341	0.2693	1.265	0.206	-0.187	0.868
**Weber et al., 2022**	0.018	0.0400	0.444	0.657	-0.061	0.096
BETWEEN RESPONSIVENESS FOR SHEAR WAVE SPEED IMAGING						
ID	**pSMD**	**SE[a]**	**t**	**Sig. (2-tailed)**	**Pooled CI Lower**	**Pooled CI Upper**
**Alijnovic et al., 2022**	0.110	0.1532	0.720	0.471	-0.190	0.411
**Buran Cirak et al. 2021**	0.657	0.3825	1.717	0.086	-0.093	1.406
**Gunaydin et al., 2022**	0.266	0.1165	2.283	0.022	0.038	0.494
**Koppenhaver et al., 2022**	0.351	0.0626	5.602	0.000	0.228	0.474
**Valero-Calero et al., 2022**	0.177	0.2066	0.857	0.392	-0.228	0.582

a. **Knapp-Hartung method is used for standard error adjustment.** b. **Some statistics cannot be computed because this subgroup contains a single record.** pSMD: **pooled standardized mean difference;** ID: **study identification;** SE: **standard error of the pooled effect size.** CI: **confidence interval;** t: **t statistic of the pooled effect size;** Sig.: **P value associated to the t statistic.**

Table S5 a – Results of heterogeneity analysis and meta-regression for strain imaging studies.

Heterogeneity and meta-regression: strain imaging						
**Known-groups validation.** M = 12, N = 569<br>Tau^2= 0.463, I^2= 98.3, Q = 569.191, df = 11 (P < 0.001)	**F^2**	**df 1**	**df 2**	**Sig.**	**R^2**	***M***
	**Univariate meta-regression**					
**Excitation method** (body movement x manual compression)	27.575	1	10	< 0.001	76	12
**Research x clinical device**	Number of records is insufficient for meta-analysis. All studies but one used research devices.					
**Medical condition** (LBP x neck or shoulder pain)	2.569	1	8	0.141	17.3	10
**Mean age**	0.008	1	10	0.929	0	12
**BMI**	1.107	1	3	0.370	23.2	5
**% of females**	3.156	1	10	0.106	14.8	12
**Extrinsic x intrinsic**	0.254	1	10	0.625	0	12
**Position** (prone x seated x upright)	0.597	2	9	0.571	0	12
**Muscle state** (contracted x rest)	4.464	1	8	0.068	34.0	10
**Vertebral level** (cervical x lumbar)	4.287	1	10	0.065	28.1	12
**Within-group responsiveness.** M = 9, N = 302<br>Tau^2= 0.206, I^2= 90.0, Q = 109.884, df = 8 (P < 0.001)	**F^2**	**df 1**	**df 2**	**Sig.**	**R^2**	***M***
	**Univariate meta-regression**					
**Excitation method** (body movement x manual compression)	0.370	1	7	0.562	0	9
**Research x clinical device**	0.006	1	7	0.939	0	9
**Medical condition** (LBP x asymptomatic)	0.180	1	6	0.686	0	8
**Mean age**	2.947	1	7	0.130	26.3	9
**BMI**	3.183	1	4	0.149	41.2	6
**% of females**	0.272	1	6	0.621	0	8
**Extrinsic x intrinsic**	0.022	1	7	0.887	0	9
**Position** (prone x seated)	3.532	1	6	0.109	25.5	8
**Muscle state** (contracted x rest)	0.060	1	5	0.816	0	7
**Vertebral level** (cervical x lumbar)	0.803	1	7	0.400	0	9
**Time of measurements after baseline** (immediate x not immediate)	0.485	1	7	0.508	0	9
**Time of measurements after baseline** (within 48 h x after 48 h)	2.469	1	7	0.160	13.5	9
**Time of measurements after baseline** (immediate x after more than one week)	1.491	1	6	0.268	0.4	8
**Treatment** (manual therapy x others)	0.013	1	7	0.913	0	9
**Between-groups responsiveness.**<br>M = 3, number of records is insufficient for meta-analysis.	**F^2**	**df 1**	**df 2**	**Sig.**	**R^2**	**k**

Tau-squared: variance of the standardized mean difference across studies; ***I*-squared:** proportion of total variance between studies that is attributed to heterogeneity; **Q:** heterogeneity statistic; ***M*:** number of cohorts; ***N*:** number of participants; **df:** degree of freedom; **F-squared:** F statistic of the meta-regression. **Sig.:** P value associated with the F statistic; **R^2:** proportion of total variance that is explained by the variable under interest.

Table S5 b – Results of Heterogeneity analysis and meta-regression for shear wave imaging.

Heterogeneity and meta-regression: shear wave imaging						
**Known-groups validation.**<br>*M*= 22, N = 1578<br>Tau^2= 0.182, I^2= 93.2, Q= 695.162, df = 21 (P < 0.001)	**F^2**	**df 1**	**df 2**	**Sig.**	**R^2**	***M***
	**Univariate meta-regression**					
**Research x clinical device**	Number of records is insufficient for meta-analysis. All studies used clinical devices.					
**Medical condition** (LBP x neck or shoulder pain)	1.489	1	15	0.241	3.0	17
**Mean age**	0.175	1	18	0.681	0	20
**BMI**	0.000	1	9	0.989	0	11
**% of females**	0.042	1	17	0.840	0	19
**Extrinsic x intrinsic**	1.289	1	18	0.271	0	20
**Position** (prone x seated)	3.951	1	20	0.061	12	20
**Muscle state** (contracted x rest x other)	Number of records is insufficient for meta-analysis. Studies were conducted only at rest.					
**Vertebral level** (cervical x lumbar)	3.473	1	16	0.081	12.9	18
**Within-group responsiveness.**<br>*M* = 12, N = 390<br>Tau^2= 1.219, I^2= 99.3 Q= 605.118, df = 11 (P < 0.001)	**F^2**	**df 1**	**df 2**	**Sig.**	**R^2**	***M***
	**Univariate meta-regression**					
**Research x clinical device**	Number of records is insufficient for meta-analysis. All studies used clinical devices.					
**Medical condition** (neck or shoulder pain x asymptomatic)	0.945	1	5	0.376	0	7
**Mean age**	0.801	1	9	0.394	0.5	11
**BMI**	0.300	1	3	0.622	0	5
**% of females**	0.072	1	7	0.796	0	9
**Extrinsic x intrinsic**	0.097	1	10	0.762	0	12
**Position** (prone x seated)	0.520	1	9	0.489	0	11
**Muscle state** (contracted x rest)	Number of records is insufficient for meta-analysis. Studies were conducted only at rest.					
**Vertebral level** (cervical x lumbar)	1.687	2	9	0.239	9.5	12
**Time of measurements after baseline** (immediate x not immediate)	0.442	1	10	0.521	0	12
**Time of measurements after baseline** (within 24 h x after 24 h)	0.089	1	10	0.772	0	12
**Time of measurements after baseline** (within 48 h x after 48 h)	0.011	1	10	0.917	0	12
**Time of measurements after baseline** (immediate x within 1 month)	0.624	1	9	0.450	0	11
**Treatment** (manual therapy x other)	0.134	1	10	0.722	0	12
**Treatment** (active physical exercise x other)	0.288	1	10	0.603	0	12
**Between-groups responsiveness.**	**F^2**	**df 1**	**df 2**	**Sig.**	**R^2**	***M***
M = 5, number of records is insufficient for meta-analysis.						

Tau-squared: variance of the standardized mean difference across studies; ***I*-squared:** proportion of total variance between studies that is attributed to heterogeneity; **Q:** heterogeneity statistic; ***M*:** number of cohorts; ***N*:** number of participants; **df:** degree of freedom; **F-squared:** F statistic of the meta-regression **Sig.:** P value associated with the F statistic; **R^2:** proportion of total variance that is explained by the ultrasound excitation method used.
